# Supplementary material for: The relationship between psychosocial circumstances and injuries in adolescents: An analysis of 87,269 individuals from 26 countries using the Global School-based Student Health Survey
Source: PLoS Med. 2021 Sep 28;18(9):e1003722. doi: 10.1371/journal.pmed.1003722 (PMC8478259; doi:10.1371/journal.pmed.1003722)
Supplement: S1 STROBE Checklist — (PDF) [file pmed.1003722.s001.pdf]

STROBE Statement—checklist of items that should be included in reports of observational studies

	Item No	Recommendation	Page No.
**Title and abstract**	1	(*a*) Indicate the study's design with a commonly used term in the title or the abstract	Title. Abstract (Methods and Findings)
		(*b*) Provide in the abstract an informative and balanced summary of what was done and what was found	Abstract (Methods and Findings; all paragraphs)
**Introduction**			
Background/rationale	2	Explain the scientific background and rationale for the investigation being reported	Abstract (Background), Author's summary (Why was this study done?), Introduction (all paragraphs)
Objectives	3	State specific objectives, including any prespecified hypotheses	Abstract (Background), Author's summary (Why was this study done?), Introduction (paragraph 4)
**Methods**			
Study design	4	Present key elements of study design early in the paper	Methods (all paragraphs)
Setting	5	Describe the setting, locations, and relevant dates, including periods of recruitment, exposure, follow-up, and data collection	Methods (paragraph 1 and 2), Appendix Table 2
Participants	6	(*a*) *Cohort study*—Give the eligibility criteria, and the sources and methods of selection of participants. Describe methods of follow-up<br>*Case-control study*—Give the eligibility criteria, and the sources and methods of case ascertainment and control selection. Give the rationale for the choice of cases and controls<br>*Cross-sectional study*—Give the eligibility criteria, and the sources and methods of selection of participants	Methods (paragraph 1, 2 and 8)
		(*b*) *Cohort study*—For matched studies, give matching criteria and number of exposed and unexposed<br>*Case-control study*—For matched studies, give matching criteria and the number of controls per case	
Variables	7	Clearly define all outcomes, exposures, predictors, potential confounders, and effect modifiers. Give diagnostic criteria, if applicable	Methods (paragraphs 3 to 9), Panel 1, Appendix Table 1
Data sources/ measurement	8*	For each variable of interest, give sources of data and details of methods of assessment (measurement). Describe	Method (paragraphs 1 to 9), Panel 1,

		comparability of assessment methods if there is more than one group	Appendix Table 1
Bias	9	Describe any efforts to address potential sources of bias	This is a secondary analysis of previously collected data. Sources of bias are explored in the limitations section; Discussion (paragraphs 11 and 12)
Study size	10	Explain how the study size was arrived at	This is a secondary analysis of previously collected data, no sample size calculation was appropriate for this analysis
Quantitative variables	11	Explain how quantitative variables were handled in the analyses. If applicable, describe which groupings were chosen and why	All variables were categorical.
Statistical methods	12	(*a*) Describe all statistical methods, including those used to control for confounding	Methods (paragraphs 10 to 14)
		(*b*) Describe any methods used to examine subgroups and interactions	NA
		(*c*) Explain how missing data were addressed	Methods (paragraph 14)
		(*d*) *Cohort study*—If applicable, explain how loss to follow-up was addressed<br>*Case-control study*—If applicable, explain how matching of cases and controls was addressed<br>*Cross-sectional study*—If applicable, describe analytical methods taking account of sampling strategy	Methods (paragraph 10)
		(*e*) Describe any sensitivity analyses	Methods (paragraph 11)
**Results**			
Participants	13*	(a) Report numbers of individuals at each stage of study—eg numbers potentially eligible, examined for eligibility, confirmed eligible, included in the study, completing follow-up, and analysed	Results (paragraph 1), Table 1, Appendix Table 2
		(b) Give reasons for non-participation at each stage	NA
		(c) Consider use of a flow diagram	NA
Descriptive data	14*	(a) Give characteristics of study participants (eg demographic, clinical, social) and information on exposures and potential confounders	Table 1, Appendix Table 3, Appendix Table 5a-d
		(b) Indicate number of participants with missing data for each variable of interest	NA
		(c) *Cohort study*—Summarise follow-up time (eg, average	

		and total amount)	
Outcome data	15*	*Cohort study*—Report numbers of outcome events or summary measures over time	
		Case-control study—Report numbers in each exposure category, or summary measures of exposure	
		Cross-sectional study—Report numbers of outcome events or summary measures	Results (paragraph 2), Table 1, Table 3
Main results	16	(*a*) Give unadjusted estimates and, if applicable, confounder-adjusted estimates and their precision (eg, 95% confidence interval). Make clear which confounders were adjusted for and why they were included	Results (paragraphs 3-7, 8-15), Tables 2, 4 and 5, Appendix Table 4, Figures 1 and 2 and Appendix Figure 1
		(*b*) Report category boundaries when continuous variables were categorized	NA
		(*c*) If relevant, consider translating estimates of relative risk into absolute risk for a meaningful time period	NA
Other analyses	17	Report other analyses done—eg analyses of subgroups and interactions, and sensitivity analyses	Results (paragraph 7), Table 2, Appendix Table 4
Discussion			
Key results	18	Summarise key results with reference to study objectives	Discussion (paragraphs 1-10, 14-15)
Limitations	19	Discuss limitations of the study, taking into account sources of potential bias or imprecision. Discuss both direction and magnitude of any potential bias	Discussion (paragraph 11-13
Interpretation	20	Give a cautious overall interpretation of results considering objectives, limitations, multiplicity of analyses, results from similar studies, and other relevant evidence	Discussion (throughout and paragraphs 14-15)
Generalisability	21	Discuss the generalisability (external validity) of the study results	Discussion (paragraph 15)
Other information			
Funding	22	Give the source of funding and the role of the funders for the present study and, if applicable, for the original study on which the present article is based	Funding Statement

*Give information separately for cases and controls in case-control studies and, if applicable, for exposed and unexposed groups in cohort and cross-sectional studies.

Note: An Explanation and Elaboration article discusses each checklist item and gives methodological background and published examples of transparent reporting. The STROBE checklist is best used in conjunction with this article (freely available on the Web sites of PLoS Medicine at http://www.plosmedicine.org/, Annals of Internal Medicine at http://www.annals.org/, and Epidemiology at http://www.epidem.com/). Information on the STROBE Initiative is available at www.strobe-statement.org.
