# Supplementary figures and images for: The relationship between psychosocial circumstances and injuries in adolescents: An analysis of 87,269 individuals from 26 countries using the Global School-based Student Health Survey
Source: PLoS Med. 2021 Sep 28;18(9):e1003722. doi: 10.1371/journal.pmed.1003722 (PMC8478259; doi:10.1371/journal.pmed.1003722)

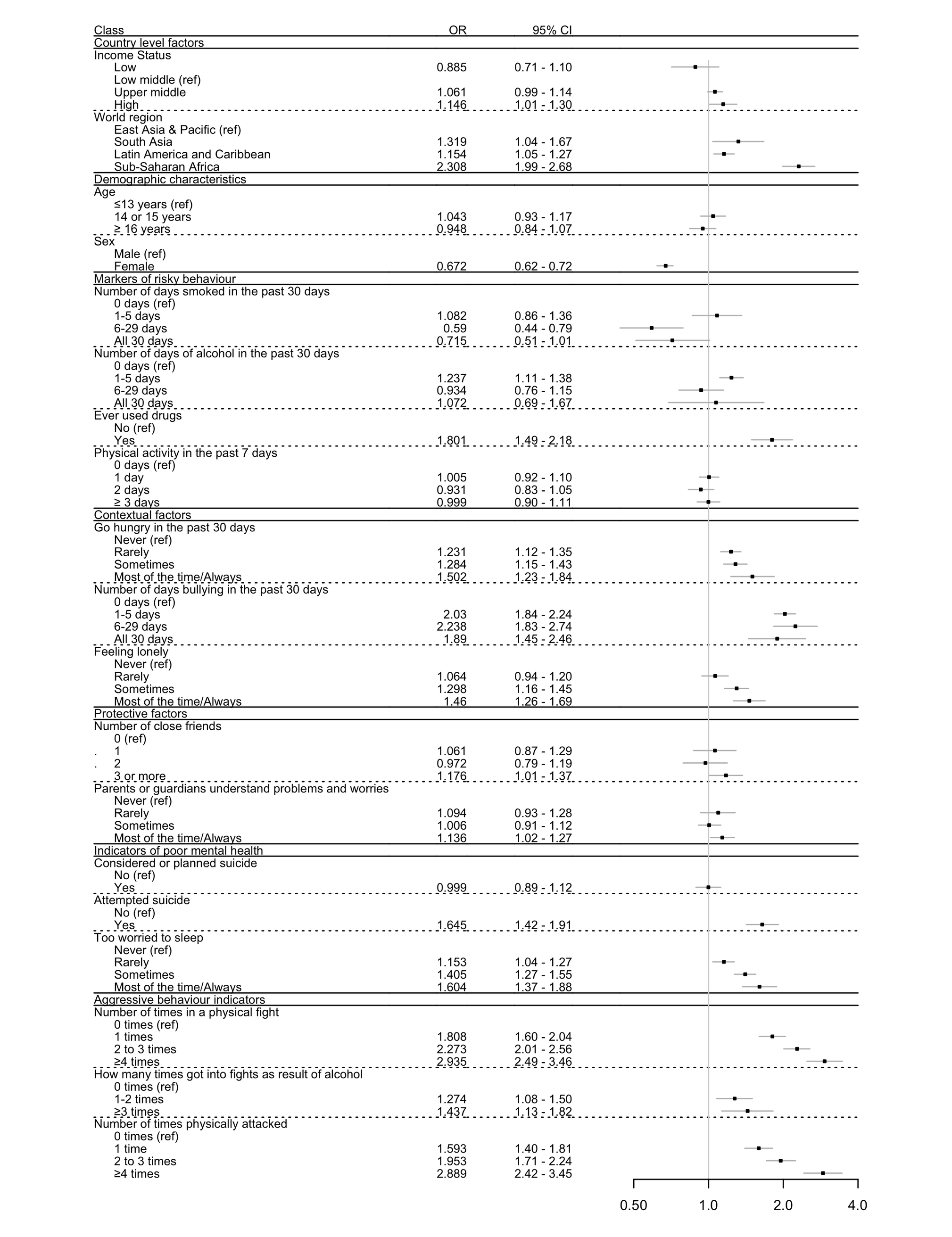

Supplement: S1 Fig — Adjustment for non-response rate of participants was done as described in Methods. p-Values can be found in Table 3 (Model 2). (TIF) [file pmed.1003722.s002.tif]
