# Supplementary material for: The relationship between psychosocial circumstances and injuries in adolescents: An analysis of 87,269 individuals from 26 countries using the Global School-based Student Health Survey
Source: PLoS Med. 2021 Sep 28;18(9):e1003722. doi: 10.1371/journal.pmed.1003722 (PMC8478259; doi:10.1371/journal.pmed.1003722)
Supplement: S1 Text — Table A: Number of responses of the total population and by occurrence of any serious injury in the last 12 months, for each of our variables for original and combined survey questions. Combined responses are shown highlighted yellow or orange. Table B: Characteristics of included countries and their populations (unweighted, unless stated otherwise). *Bangladesh did not have data on mechanism of injury, so was excluded from those analyses, but included in all others. Table C: Characteristics of the total population and by occurrence of any serious injury in the last 12 months, in total and by sex. Weights take into account the 2-stage study design and are also adjusted for the non-response rate of participants; see Methods for a full explanation. Table D: Multivariate analysis showing association between serious injury occurrence and individual characteristics, including aggressive behaviour indicators (Model 1), with income status (Model 2) and world region (Model 3) added separately. Table E: Characteristics of the total population and by occurrence of any serious injury in the last 12 months, in total:—Complete cases used in analysis of type of injury. Weights take into account the 2-stage study design and are also adjusted for the non-response rate of participants; see Methods for a full explanation. Table F: Characteristics of the total population and by occurrence of any serious injury in the last 12 months, by sex: Complete cases used in analysis of type of injury. Weights take into account the 2-stage study design and are also adjusted for the non-response rate of participants; see Methods for a full explanation. Table G: Characteristics of the total population and by occurrence of any serious injury in the last 12 months, in total: Complete cases used in analysis of mechanism of injury. Weights take into account the 2-stage study design and are also adjusted for the non-response rate of participants; see Methods for a full explanation. *Data on mechanism of [file pmed.1003722.s003.docx]

Table A in S1 Text. Number of responses of the total population and by occurrence of any serious injury in the last 12 months, for each of our variables for original and combined survey questions. Combined responses are shown highlighted as yellow or orange.

| Total number of responses: | 87269 |  |  |  |  |  |  |
| --- | --- | --- | --- | --- | --- | --- | --- |
| **Orignal questions** | **Total (%)** | **No Serious Injuries (%)** | **≥1 Serious Injury (%)** | **Combined questions used in study** | **Total (%)** | **No Serious Injuries (%)** | **≥1 Serious Injury(%)** |
| **During the past 30 days, on how many days did you smoke cigarettes?** | | |  | **Number of days smoked in the past 30 days** | | |  |
| 0 days | 76958 (88) | 51294 (59) | 25664 (29) | Never (0 days) | 76958 (88) | 51294 (59) | 25664 (29) |
| 1 or 2 days | 4651 (5) | 2340 (3) | 2311 (3) | Rarely (1 or 2/3-5 days) | 6167 (7) | 3074 (4) | 3093 (4) |
| 3 to 5 days | 1516 (2) | 734 (1) | 782 (1) |  |  |  |  |
| 6 to 9 days | 916 (1) | 448 (1) | 468 (1) | Often (6-9/10-19/20-29 days) | 2476 (3) | 1278 (1) | 1198 (1) |
| 10 to 19 days | 1008 (1) | 555 (1) | 453 (1) |  |  |  |  |
| 20 to 29 days | 552 (1) | 275 (0) | 277 (0) |  |  |  |  |
| All 30 days | 1668 (2) | 832 (1) | 836 (1) | Always (all 30 days) | 1668 (2) | 832 (1) | 836 (1) |
| **During the past 30 days, on how many days did you have at least one drink containing alcohol?** | | | | **Number of days of alcohol in the past 30 days** | | |  |
| 0 days | 67317 (77) | 45293 (52) | 22024 (25) | Never (0 days) | 67317 (77) | 45293 (52) | 22024 (25) |
| 1 or 2 days | 12152 (14) | 7046 (8) | 5106 (6) | Rarely (1 or 2/3-5 days) | 15729 (18) | 9027 (10) | 6702 (8) |
| 3 to 5 days | 3577 (4) | 1981 (2) | 1596 (2) |  |  |  |  |
| 6 to 9 days | 1902 (2) | 1007 (1) | 895 (1) | Often (6-9/10-19/20-29 days) | 3664 (4) | 1891 (2) | 1773 (2) |
| 10 to 19 days | 1286 (1) | 650 (1) | 636 (1) |  |  |  |  |
| 20 to 29 days | 476 (1) | 234 (0) | 242 (0) |  |  |  |  |
| All 30 days | 559 (1) | 267 (0) | 292 (0) | Always (all 30 days) | 559 (1) | 267 (0) | 292 (0) |
| **How old were you when you first used drugs?** | |  |  | **Ever used drugs** |  |  |  |
| I have never used drugs | 81497 (93) | 53967 (62) | 27530 (32) | No | 81497 (93) | 53967 (62) | 27530 (32) |
| 7 years old or younger | 850 (1) | 285 (0) | 565 (1) | Yes | 5772 (7) | 2511 (3) | 3261 (4) |
| 8 or 9 years old | 449 (1) | 141 (0) | 308 (0) |  |  |  |  |
| 10 or 11 years old | 481 (1) | 185 (0) | 296 (0) |  |  |  |  |
| 12 or 13 years old | 1259 (1) | 559 (1) | 700 (1) |  |  |  |  |
| 14 or 15 years old | 1874 (2) | 913 (1) | 961 (1) |  |  |  |  |
| 16 years old or older | 455 (1) | 229 (0) | 226 (0) |  |  |  |  |
| 16 or 17 years old | 299 (0) | 147 (0) | 152 (0) |  |  |  |  |
| 18 years old or older | 105 (0) | 52 (0) | 53 (0) |  |  |  |  |
| **During the past 7 days, on how many days were you physically active for a total of at least 60 minutes per day?** | | | | **Physical activity in the past 7 days** | |  |  |
| 0 days | 19470 (22) | 12960 (15) | 6510 (7) | 0 days | 19470 (22) | 12960 (15) | 6510 (7) |
| 1 day | 17689 (20) | 11877 (14) | 5812 (7) | 1 day | 17689 (20) | 11877 (14) | 5812 (7) |
| 2 days | 13049 (15) | 8831 (10) | 4218 (5) | 2 days | 13049 (15) | 8831 (10) | 4218 (5) |
| 3 days | 9312 (11) | 5922 (7) | 3390 (4) | ≥ 3 days | 37061 (42) | 22810 (26) | 14251 (16) |
| 4 days | 5342 (6) | 3357 (4) | 1985 (2) |  |  |  |  |
| 5 days | 5368 (6) | 3206 (4) | 2162 (2) |  |  |  |  |
| 6 days | 2494 (3) | 1412 (2) | 1082 (1) |  |  |  |  |
| 7 days | 14545 (17) | 8913 (10) | 5632 (6) |  |  |  |  |
| **During the past 30 days, how often did you go hungry because there was not enough food in your home?** | | | | **Go hungry in the past 30 days** | |  |  |
| Never | 43236 (50) | 30558 (35) | 12678 (15) | Never | 43236 (50) | 30558 (35) | 12678 (15) |
| Rarely | 18743 (21) | 11655 (13) | 7088 (8) | Rarely | 18743 (21) | 11655 (13) | 7088 (8) |
| Sometimes | 20764 (24) | 12074 (14) | 8690 (10) | Sometimes | 20764 (24) | 12074 (14) | 8690 (10) |
| Most of the time | 2813 (3) | 1397 (2) | 1416 (2) | Most of the time/always | 4526 (5) | 2191 (3) | 2335 (3) |
| Always | 1713 (2) | 794 (1) | 919 (1) |  |  |  |  |
| **During the past 30 days, on how many days were you bullied?** | | |  | **Number of days bullying in the past 30 days** | | |  |
| 0 days | 65734 (75) | 46831 (54) | 18903 (22) | Never (0 days) | 65734 (75) | 46831 (54) | 18903 (22) |
| 1 or 2 days | 13564 (16) | 6461 (7) | 7103 (8) | Rarely (1 or 2/3-5 days) | 17251 (20) | 7964 (9) | 9287 (11) |
| 3 to 5 days | 3687 (4) | 1503 (2) | 2184 (3) |  |  |  |  |
| 6 to 9 days | 1462 (2) | 548 (1) | 914 (1) | Often (6-9/10-19/20-29 days) | 2853 (3) | 1089 (1) | 1764 (2) |
| 10 to 19 days | 943 (1) | 367 (0) | 576 (1) |  |  |  |  |
| 20 to 29 days | 448 (1) | 174 (0) | 274 (0) |  |  |  |  |
| All 30 days | 1431 (2) | 594 (1) | 837 (1) | Always (all 30 days) | 1431 (2) | 594 (1) | 837 (1) |
| **During the past 12 months, how often have you felt lonely?** | | |  | **Feeling lonely** |  |  |  |
| Never | 30343 (35) | 21663 (25) | 8680 (10) | Never | 30343 (35) | 21663 (25) | 8680 (10) |
| Rarely | 21858 (25) | 14376 (16) | 7482 (9) | Rarely | 21858 (25) | 14376 (16) | 7482 (9) |
| Sometimes | 26694 (31) | 16158 (19) | 10536 (12) | Sometimes | 26694 (31) | 16158 (19) | 10536 (12) |
| Most of the time | 5952 (7) | 3138 (4) | 2814 (3) | Most of the time/Always | 8374 (10) | 4281 (5) | 4093 (5) |
| Always | 2422 (3) | 1143 (1) | 1279 (1) |  |  |  |  |
| **How many close friends do you have?** | |  |  | **Number of close friends** |  |  |  |
| 0 | 4355 (5) | 2651 (3) | 1704 (2) | 0 | 4355 (5) | 2651 (3) | 1704 (2) |
| 1 | 8677 (10) | 5374 (6) | 3303 (4) | 1 | 8677 (10) | 5374 (6) | 3303 (4) |
| 2 | 11071 (13) | 6938 (8) | 4133 (5) | 2 | 11071 (13) | 6938 (8) | 4133 (5) |
| 3 or more | 63166 (72) | 41515 (48) | 21651 (25) | 3 or more | 63166 (72) | 41515 (48) | 21651 (25) |
| **During the past 30 days, how often did your parents or guardians understand your problems and worries?** | | | | **Parents or guardians understand problems and worries** | | |  |
| Never | 18358 (21) | 11923 (14) | 6435 (7) | Never | 18358 (21) | 11923 (14) | 6435 (7) |
| Rarely | 14383 (16) | 9020 (10) | 5363 (6) | Rarely | 14383 (16) | 9020 (10) | 5363 (6) |
| Sometimes | 21145 (24) | 13337 (15) | 7808 (9) | Sometimes | 21145 (24) | 13337 (15) | 7808 (9) |
| Most of the time | 13803 (16) | 8895 (10) | 4908 (6) | Most of the time/Always | 33383 (38) | 22198 (25) | 11185 (13) |
| Always | 19580 (22) | 13303 (15) | 6277 (7) |  |  |  |  |
| **During the past 12 months, did you ever seriously consider attempting suicide?** | | | | **Considered or planned suicide it answer yes to either question** | | |  |
| No | 76923 (88) | 51393 (59) | 25530 (29) | No | 73710 (84) | 49729 (57) | 23981 (27) |
| Yes | 10346 (12) | 5085 (6) | 5261 (6) | Yes | 13559 (16) | 6749 (8) | 6810 (8) |
| **During the past 12 months, did you make a plan about how you would attempt suicide?** | | | |  |  |  |  |
| No | 77868 (89) | 51922 (59) | 25946 (30) |  |  |  |  |
| Yes | 9401 (11) | 4556 (5) | 4845 (6) |  |  |  |  |
| **During the past 12 months, how many times did you actually attempt suicide?** | | | | **Attempted suicide** |  |  |  |
| 0 times | 78359 (90) | 52566 (60) | 25793 (30) | No | 78359 (90) | 52566 (60) | 25793 (30) |
| 1 time | 5496 (6) | 2654 (3) | 2842 (3) | Yes | 8910 (10) | 3912 (4) | 4998 (6) |
| 2 or 3 times | 2140 (2) | 799 (1) | 1341 (2) |  |  |  |  |
| 4 or 5 times | 664 (1) | 268 (0) | 396 (0) |  |  |  |  |
| 6 or more times | 610 (1) | 191 (0) | 419 (0) |  |  |  |  |
| **During the past 12 months, how often have you been so worried about something that you could not sleep at night?** | | | | **Too worried to sleep** |  |  |  |
| Never | 33966 (39) | 24713 (28) | 9253 (11) | Never | 33966 (39) | 24713 (28) | 9253 (11) |
| Rarely | 24288 (28) | 15770 (18) | 8518 (10) | Rarely | 24288 (28) | 15770 (18) | 8518 (10) |
| Sometimes | 22817 (26) | 13146 (15) | 9671 (11) | Sometimes | 22817 (26) | 13146 (15) | 9671 (11) |
| Most of the time | 4516 (5) | 2147 (2) | 2369 (3) | Most of the time/Always | 6198 (7) | 2849 (3) | 3349 (4) |
| Always | 1682 (2) | 702 (1) | 980 (1) |  |  |  |  |
| **During the past 12 months, how many times were you in a physical fight?** | | |  | **Number of times in a physical fight** | |  |  |
| 0 times | 63386 (73) | 45747 (52) | 17639 (20) | 0 times | 63386 (73) | 45747 (52) | 17639 (20) |
| 1 time | 11279 (13) | 5789 (7) | 5490 (6) | 1 times | 11279 (13) | 5789 (7) | 5490 (6) |
| 2 or 3 times | 7291 (8) | 3160 (4) | 4131 (5) | 2 to 3 times | 7291 (8) | 3160 (4) | 4131 (5) |
| 4 or 5 times | 2089 (2) | 740 (1) | 1349 (2) | ≥4 times | 5313 (6) | 1782 (2) | 3531 (4) |
| 6 or 7 times | 880 (1) | 295 (0) | 585 (1) |  |  |  |  |
| 8 or 9 times | 433 (0) | 134 (0) | 299 (0) |  |  |  |  |
| 10 or 11 times | 259 (0) | 76 (0) | 183 (0) |  |  |  |  |
| 12 or more times | 1652 (2) | 537 (1) | 1115 (1) |  |  |  |  |
| **During your life, how many times have you got into trouble with your family or friends, missed school, or got into fights, as a result of drinking alcohol?** | | | | **How many times got into fights as result of alcohol** | | |  |
| 0 times | 79740 (91) | 52954 (61) | 26786 (31) | 0 times | 79740 (91) | 52954 (61) | 26786 (31) |
| 1 or 2 times | 5315 (6) | 2637 (3) | 2678 (3) | 1 to 2 times | 5315 (6) | 2637 (3) | 2678 (3) |
| 3 to 9 times | 1329 (2) | 556 (1) | 773 (1) | >3 times | 2214 (3) | 887 (1) | 1327 (2) |
| 10 or more times | 885 (1) | 331 (0) | 554 (1) |  |  |  |  |
| **During the past 12 months, how many times were you physically attacked?** | | | | **Number of times physically attacked** | |  |  |
| 0 times | 63807 (73) | 45927 (53) | 17880 (20) | 0 times | 63807 (73) | 45927 (53) | 17880 (20) |
| 1 time | 9684 (11) | 4929 (6) | 4755 (5) | 1 times | 9684 (11) | 4929 (6) | 4755 (5) |
| 2 or 3 times | 7760 (9) | 3493 (4) | 4267 (5) | 2 to 3 times | 7760 (9) | 3493 (4) | 4267 (5) |
| 4 or 5 times | 2269 (3) | 868 (1) | 1401 (2) | ≥4 | 6018 (7) | 2129 (2) | 3889 (4) |
| 6 or 7 times | 989 (1) | 333 (0) | 656 (1) |  |  |  |  |
| 8 or 9 times | 576 (1) | 197 (0) | 379 (0) |  |  |  |  |
| 10 or 11 times | 350 (0) | 101 (0) | 249 (0) |  |  |  |  |
| 12 or more times | 1834 (2) | 630 (1) | 1204 (1) |  |  |  |  |

Table B in S1 Text. Characteristics of included countries and their populations (unweighted, unless stated otherwise). *Bangladesh did not have data on mechanism of injury, so was excluded form those analyses, but included in all others

| Country | Survey year | World Bank Geographical Region | Income status | Survey Weighted  Prevelance | Sample Size | Serious Injuries,  n (%) | Sex, n (%) | | Age categories, n (%) | | |
| --- | --- | --- | --- | --- | --- | --- | --- | --- | --- | --- | --- |
|  |  |  |  |  |  |  | Male | Female | ≤13 years | 14 or 15 years | ≥ 16 years |
| Argentina | 2012 | Latin America & Caribbean | Upper middle income | 3.65% | 18347 | 5997 (33%) | 8586 (47%) | 9761 (53%) | 3739 (20%) | 10418 (57%) | 4190 (23%) |
| Bahamas | 2013 | Latin America & Caribbean | High income | 0.04% | 840 | 348 (41%) | 367 (44%) | 473 (56%) | 474 (56%) | 350 (42%) | 16 (2%) |
| Bangladesh* | 2014 | South Asia | Lower middle income | 15.55% | 2198 | 731 (33%) | 916 (42%) | 1282 (58%) | 512 (23%) | 1528 (70%) | 158 (7%) |
| Benin | 2010 | Sub-Saharan Africa | Low income | 0.52% | 1632 | 811 (50%) | 1107 (68%) | 525 (32%) | 143 (9%) | 580 (36%) | 909 (56%) |
| Bolivia | 2012 | Latin America & Caribbean | Lower middle income | 1.48% | 2239 | 1019 (46%) | 1141 (51%) | 1098 (49%) | 538 (24%) | 1266 (57%) | 435 (19%) |
| British Virgin Islands | 2009 | Latin America & Caribbean | High income | 0.01% | 1137 | 474 (42%) | 492 (43%) | 645 (57%) | 432 (38%) | 440 (39%) | 265 (23%) |
| Brunei Darussalam | 2014 | East Asia & Pacific | High income | 0.08% | 1967 | 531 (27%) | 887 (45%) | 1080 (55%) | 479 (24%) | 905 (46%) | 583 (30%) |
| Cook Islands | 2015 | East Asia & Pacific | High income | 0.00% | 502 | 259 (52%) | 240 (48%) | 262 (52%) | 42 (8%) | 206 (41%) | 254 (51%) |
| Costa Rica | 2009 | Latin America & Caribbean | Upper middle income | 0.72% | 2058 | 448 (22%) | 968 (47%) | 1090 (53%) | 603 (29%) | 1145 (56%) | 310 (15%) |
| Curaçao | 2015 | Latin America & Caribbean | High income | 0.03% | 2003 | 573 (29%) | 849 (42%) | 1154 (58%) | 388 (19%) | 656 (33%) | 959 (48%) |
| Ghana | 2012 | Sub-Saharan Africa | Lower middle income | 4.86% | 2060 | 1165 (57%) | 1169 (57%) | 891 (43%) | 254 (12%) | 505 (25%) | 1301 (63%) |
| Indonesia | 2015 | East Asia & Pacific | Lower middle income | 37.74% | 8312 | 2178 (26%) | 3548 (43%) | 4764 (57%) | 3290 (40%) | 3400 (41%) | 1622 (20%) |
| Kiribati | 2011 | East Asia & Pacific | Lower middle income | 0.01% | 865 | 447 (52%) | 365 (42%) | 500 (58%) | 220 (25%) | 517 (60%) | 128 (15%) |
| Lao People's Democratic Republic | 2015 | East Asia & Pacific | Lower middle income | 0.93% | 2838 | 456 (16%) | 1287 (45%) | 1551 (55%) | 146 (5%) | 1121 (39%) | 1571 (55%) |
| Malaysia | 2012 | East Asia & Pacific | Upper middle income | 6.70% | 18879 | 5907 (31%) | 9022 (48%) | 9857 (52%) | 3920 (21%) | 8018 (42%) | 6941 (37%) |
| Mongolia | 2013 | East Asia & Pacific | Lower middle income | 0.78% | 4119 | 1422 (35%) | 1883 (46%) | 2236 (54%) | 1369 (33%) | 1505 (37%) | 1245 (30%) |
| Mozambique | 2015 | Sub-Saharan Africa | Low income | 1.67% | 1012 | 473 (47%) | 539 (53%) | 473 (47%) | 95 (9%) | 281 (28%) | 636 (63%) |
| Namibia | 2013 | Sub-Saharan Africa | Upper middle income | 0.57% | 2660 | 1286 (48%) | 1267 (48%) | 1393 (52%) | 338 (13%) | 781 (29%) | 1541 (58%) |
| Peru | 2011 | Latin America & Caribbean | Upper middle income | 3.81% | 2225 | 1053 (47%) | 1067 (48%) | 1158 (52%) | 438 (20%) | 1406 (63%) | 381 (17%) |
| Philippines | 2015 | East Asia & Pacific | Lower middle income | 20.50% | 6233 | 2788 (45%) | 2729 (44%) | 3504 (56%) | 1656 (27%) | 2800 (45%) | 1777 (29%) |
| Samoa | 2011 | East Asia & Pacific | Upper middle income | 0.01% | 639 | 466 (73%) | 215 (34%) | 424 (66%) | 175 (27%) | 429 (67%) | 35 (5%) |
| Solomon Islands | 2011 | East Asia & Pacific | Lower middle income | 0.04% | 658 | 412 (63%) | 357 (54%) | 301 (46%) | 144 (22%) | 328 (50%) | 186 (28%) |
| Suriname | 2009 | Latin America & Caribbean | Upper middle income | 0.06% | 1034 | 308 (30%) | 503 (49%) | 531 (51%) | 226 (22%) | 432 (42%) | 376 (36%) |
| Trinidad and Tobago | 2011 | Latin America & Caribbean | High income | 0.19% | 1832 | 739 (40%) | 978 (53%) | 854 (47%) | 901 (49%) | 767 (42%) | 164 (9%) |
| Tuvalu | 2013 | East Asia & Pacific | Upper middle income | 0.00% | 495 | 210 (42%) | 206 (42%) | 289 (58%) | 215 (43%) | 156 (32%) | 124 (25%) |
| Vanuatu | 2011 | East Asia & Pacific | Lower middle income | 0.03% | 485 | 290 (60%) | 207 (43%) | 278 (57%) | 211 (44%) | 195 (40%) | 79 (16%) |

Table C in S1 Text. Characteristics of the total population and by occurrence of any serious injury in the last 12 months, in total and by sex. Weights take into account the 2-stage study design and are also adjusted for the non-response rate of participants, see the methods section for a full explanation

|  | **Total** | | | | **No serious Injuries** | | | | **≥1 Serious Injury** | | | |
| --- | --- | --- | --- | --- | --- | --- | --- | --- | --- | --- | --- | --- |
|  | **(Unweighted N)** | **(Unweighted N)** | **(Weighted %)** | **(Weighted %)** | **(Unweighted N)** | **(Unweighted N)** | **(Weighted %)** | **(Weighted %)** | **(Unweighted N)** | **(Unweighted N)** | **(Weighted %)** | **(Weighted %)** |
|  | **Male** | **Female** | **Male** | **Female** | **Male** | **Female** | **Male** | **Female** | **Male** | **Female** | **Male** | **Female** |
| **Injured** | **40895** | **46374** | **50.70** | **49.30** | **23769** | **32709** | **45.69** | **54.31** | **17126** | **13665** | **59.47** | **40.53** |
| **Income Status** |  |  |  |  |  |  |  |  |  |  |  |  |
| Low | 1646 | 998 | 57.62 | 42.38 | 807 | 553 | 55.62 | 44.38 | 839 | 445 | 59.73 | 40.27 |
| Lower middle | 13602 | 16405 | 50.92 | 49.08 | 7853 | 11246 | 45.94 | 54.06 | 5749 | 5159 | 59.73 | 40.27 |
| Upper middle | 21834 | 24503 | 48.60 | 51.40 | 12893 | 17769 | 43.33 | 56.67 | 8941 | 6734 | 58.12 | 41.88 |
| High | 3813 | 4468 | 47.97 | 52.03 | 2216 | 3141 | 43.59 | 56.41 | 1597 | 1327 | 55.76 | 44.24 |
| **World region** |  |  |  |  |  |  |  |  |  |  |  |  |
| East Asia and Pacific | 20946 | 25046 | 46.70 | 53.30 | 12589 | 18037 | 42.03 | 57.97 | 8357 | 7009 | 56.49 | 43.51 |
| South Asia | 916 | 1282 | 66.66 | 33.34 | 583 | 884 | 62.34 | 37.66 | 333 | 398 | 72.52 | 27.48 |
| Latin America & Caribbean | 14951 | 16764 | 49.32 | 50.68 | 8683 | 12073 | 43.53 | 56.47 | 6268 | 4691 | 58.38 | 41.62 |
| Sub-Saharan Africa | 4082 | 3282 | 55.03 | 44.97 | 1914 | 1715 | 54.61 | 45.39 | 2168 | 1567 | 55.36 | 44.64 |
| **Demographic characteristics** |  |  |  |  |  |  |  |  |  |  |  |  |
| **Age** |  |  |  |  |  |  |  |  |  |  |  |  |
| ≤13 years | 9462 | 11486 | 47.43 | 52.57 | 5424 | 8192 | 41.00 | 59.00 | 4038 | 3294 | 59.80 | 40.20 |
| 14 or 15 years | 18600 | 21535 | 52.00 | 48.00 | 10768 | 15084 | 47.80 | 52.20 | 7832 | 6451 | 59.01 | 40.99 |
| ≥16 years | 12833 | 13353 | 52.48 | 47.52 | 7577 | 9433 | 48.09 | 51.91 | 5256 | 3920 | 59.98 | 40.02 |
| **Markers of risky behaviour** |  |  |  |  |  |  |  |  |  |  |  |  |
| **Number of days smoked in the past 30 days** |  |  |  |  |  |  |  |  |  |  |  |  |
| Never (0 days) | 34279 | 42679 | 47.34 | 52.66 | 20690 | 30604 | 42.48 | 57.52 | 13589 | 12075 | 56.36 | 43.64 |
| Rarely (1 or 2/3-5 days) | 3806 | 2361 | 79.37 | 20.63 | 1713 | 1361 | 79.60 | 20.40 | 2093 | 1000 | 79.15 | 20.85 |
| Often (6-9/10-19/20-29 days) | 1591 | 885 | 84.76 | 15.24 | 771 | 507 | 86.78 | 13.22 | 820 | 378 | 81.54 | 18.46 |
| Always (all 30 days) | 1219 | 449 | 90.41 | 9.59 | 595 | 237 | 93.80 | 6.20 | 624 | 212 | 86.40 | 13.60 |
| **Number of days of alcohol in the past 30 days** |  |  |  |  |  |  |  |  |  |  |  |  |
| Never (0 days) | 30761 | 36556 | 49.70 | 50.30 | 18797 | 26496 | 45.09 | 54.91 | 11964 | 10060 | 58.52 | 41.48 |
| Rarely (1 or 2/3-5 days) | 7855 | 7874 | 57.71 | 42.29 | 3964 | 5063 | 51.76 | 48.24 | 3891 | 2811 | 63.20 | 36.80 |
| Often (6-9/10-19/20-29 days) | 1931 | 1733 | 64.94 | 35.06 | 856 | 1035 | 56.72 | 43.28 | 1075 | 698 | 72.03 | 27.97 |
| Always (all 30 days) | 348 | 211 | 73.59 | 26.41 | 152 | 115 | 65.80 | 34.20 | 196 | 96 | 78.71 | 21.29 |
| **Ever used drugs** |  |  |  |  |  |  |  |  |  |  |  |  |
| No | 37386 | 44111 | 50.01 | 49.99 | 22346 | 31621 | 45.25 | 54.75 | 15040 | 12490 | 58.80 | 41.20 |
| Yes | 3509 | 2263 | 68.33 | 31.67 | 1423 | 1088 | 68.36 | 31.64 | 2086 | 1175 | 68.31 | 31.69 |
| **Physical activity in the past 7 days** |  |  |  |  |  |  |  |  |  |  |  |  |
| 0 days | 8251 | 11219 | 47.40 | 52.60 | 4912 | 8048 | 42.76 | 57.24 | 3339 | 3171 | 56.12 | 43.88 |
| 1 day | 7220 | 10469 | 44.24 | 55.76 | 4315 | 7562 | 38.81 | 61.19 | 2905 | 2907 | 54.22 | 45.78 |
| 2 days | 5413 | 7636 | 46.79 | 53.21 | 3322 | 5509 | 43.11 | 56.89 | 2091 | 2127 | 53.48 | 46.52 |
| ≥ 3 days | 20011 | 17050 | 58.41 | 41.59 | 11220 | 11590 | 53.37 | 46.63 | 8791 | 5460 | 66.45 | 33.55 |
| **Contextual factors** |  |  |  |  |  |  |  |  |  |  |  |  |
| **Go hungry in the past 30 days** |  |  |  |  |  |  |  |  |  |  |  |  |
| Never | 19609 | 23627 | 48.67 | 51.33 | 12637 | 17921 | 44.56 | 55.44 | 6972 | 5706 | 58.21 | 41.79 |
| Rarely | 9362 | 9381 | 53.42 | 46.58 | 5245 | 6410 | 49.22 | 50.78 | 4117 | 2971 | 60.26 | 39.74 |
| Sometimes | 9671 | 11093 | 50.92 | 49.08 | 4917 | 7157 | 44.81 | 55.19 | 4754 | 3936 | 59.81 | 40.19 |
| Most of the time/always | 2253 | 2273 | 55.31 | 44.69 | 970 | 1221 | 49.22 | 50.78 | 1283 | 1052 | 61.35 | 38.65 |
| **Number of days bullying in the past 30 days** |  |  |  |  |  |  |  |  |  |  |  |  |
| Never (0 days) | 30232 | 35502 | 49.16 | 50.84 | 19510 | 27321 | 44.74 | 55.26 | 10722 | 8181 | 60.49 | 39.51 |
| Rarely (1 or 2/3-5 days) | 8483 | 8768 | 54.24 | 45.76 | 3486 | 4478 | 49.70 | 50.30 | 4997 | 4290 | 57.69 | 42.31 |
| Often (6-9/10-19/20-29 days) | 1476 | 1377 | 58.88 | 41.12 | 512 | 577 | 53.12 | 46.88 | 964 | 800 | 61.92 | 38.08 |
| Always (all 30 days) | 704 | 727 | 52.21 | 47.79 | 261 | 333 | 47.45 | 52.55 | 443 | 394 | 55.62 | 44.38 |
| **Feeling lonely** |  |  |  |  |  |  |  |  |  |  |  |  |
| Never | 16705 | 13638 | 57.74 | 42.26 | 10944 | 10719 | 52.65 | 47.35 | 5761 | 2919 | 70.39 | 29.61 |
| Rarely | 10642 | 11216 | 53.41 | 46.59 | 6139 | 8237 | 48.67 | 51.33 | 4503 | 2979 | 63.28 | 36.72 |
| Sometimes | 10543 | 16151 | 44.59 | 55.41 | 5408 | 10750 | 37.92 | 62.08 | 5135 | 5401 | 54.19 | 45.81 |
| Most of the time/Always | 3005 | 5369 | 43.64 | 56.36 | 1278 | 3003 | 37.01 | 62.99 | 1727 | 2366 | 49.33 | 50.67 |
| **Protective factors** |  |  |  |  |  |  |  |  |  |  |  |  |
| **Number of close friends** |  |  |  |  |  |  |  |  |  |  |  |  |
| 0 | 2035 | 2320 | 50.33 | 49.67 | 1108 | 1543 | 46.60 | 53.40 | 927 | 777 | 55.68 | 44.32 |
| 1 | 3665 | 5012 | 50.37 | 49.63 | 2113 | 3261 | 49.92 | 50.08 | 1552 | 1751 | 51.09 | 48.91 |
| 2 | 4656 | 6415 | 52.69 | 47.31 | 2591 | 4347 | 49.29 | 50.71 | 2065 | 2068 | 58.05 | 41.95 |
| 3 or more | 30539 | 32627 | 50.44 | 49.56 | 17957 | 23558 | 44.52 | 55.48 | 12582 | 9069 | 61.29 | 38.71 |
| **Parents or guardians understand problems and worries** |  |  |  |  |  |  |  |  |  |  |  |  |
| Never | 9006 | 9352 | 55.52 | 44.48 | 5368 | 6555 | 50.73 | 49.27 | 3638 | 2797 | 64.01 | 35.99 |
| Rarely | 6622 | 7761 | 50.44 | 49.56 | 3687 | 5333 | 46.05 | 53.95 | 2935 | 2428 | 56.92 | 43.08 |
| Sometimes | 9898 | 11247 | 49.51 | 50.49 | 5555 | 7782 | 44.42 | 55.58 | 4343 | 3465 | 58.89 | 41.11 |
| Most of the time/Always | 15369 | 18014 | 49.26 | 50.74 | 9159 | 13039 | 44.03 | 55.97 | 6210 | 4975 | 58.56 | 41.44 |
| **Indicators of poor mental health** |  |  |  |  |  |  |  |  |  |  |  |  |
| **Considered or planned suicide** |  |  |  |  |  |  |  |  |  |  |  |  |
| No | 35645 | 38065 | 51.48 | 48.52 | 21589 | 28140 | 46.31 | 53.69 | 14056 | 9925 | 61.36 | 38.64 |
| Yes | 5250 | 8309 | 44.58 | 55.42 | 2180 | 4569 | 39.02 | 60.98 | 3070 | 3740 | 49.66 | 50.34 |
| **Attempted suicide** |  |  |  |  |  |  |  |  |  |  |  |  |
| No | 37296 | 41063 | 51.08 | 48.92 | 22457 | 30109 | 45.98 | 54.02 | 14839 | 10954 | 60.88 | 39.12 |
| Yes | 3599 | 5311 | 46.04 | 53.96 | 1312 | 2600 | 39.73 | 60.27 | 2287 | 2711 | 49.95 | 50.05 |
| **Too worried to sleep** |  |  |  |  |  |  |  |  |  |  |  |  |
| Never | 18349 | 15617 | 55.64 | 44.36 | 12301 | 12412 | 50.72 | 49.28 | 6048 | 3205 | 68.09 | 31.91 |
| Rarely | 11086 | 13202 | 50.13 | 49.87 | 6178 | 9592 | 45.28 | 54.72 | 4908 | 3610 | 59.10 | 40.90 |
| Sometimes | 9144 | 13673 | 46.15 | 53.85 | 4419 | 8727 | 39.23 | 60.77 | 4725 | 4946 | 55.21 | 44.79 |
| Most of the time/Always | 2316 | 3882 | 44.34 | 55.66 | 871 | 1978 | 37.95 | 62.05 | 1445 | 1904 | 49.22 | 50.78 |
| **Agreesive behaviour indicators** |  |  |  |  |  |  |  |  |  |  |  |  |
| **Number of times in a physical fight** |  |  |  |  |  |  |  |  |  |  |  |  |
| 0 times | 26028 | 37358 | 45.34 | 54.66 | 17306 | 28441 | 41.79 | 58.21 | 8722 | 8917 | 54.36 | 45.64 |
| 1 times | 6674 | 4605 | 63.84 | 36.16 | 3332 | 2457 | 64.72 | 35.28 | 3342 | 2148 | 63.04 | 36.96 |
| 2 to 3 times | 4602 | 2689 | 66.80 | 33.20 | 1957 | 1203 | 67.74 | 32.26 | 2645 | 1486 | 66.27 | 33.73 |
| ≥4 times | 3591 | 1722 | 72.42 | 27.58 | 1174 | 608 | 70.24 | 29.76 | 2417 | 1114 | 73.21 | 26.79 |
| **How many times got into fights as result of alcohol** |  |  |  |  |  |  |  |  |  |  |  |  |
| 0 times | 36928 | 42812 | 50.20 | 49.80 | 22146 | 30808 | 45.37 | 54.63 | 14782 | 12004 | 59.15 | 40.85 |
| 1 to 2 times | 2751 | 2564 | 59.71 | 40.29 | 1186 | 1451 | 56.20 | 43.80 | 1565 | 1113 | 62.12 | 37.88 |
| >3 times | 1216 | 998 | 63.78 | 36.22 | 437 | 450 | 60.12 | 39.88 | 779 | 548 | 65.32 | 34.68 |
| **Number of times physically attacked** |  |  |  |  |  |  |  |  |  |  |  |  |
| 0 times | 27861 | 35946 | 45.25 | 54.75 | 18325 | 27602 | 41.89 | 58.11 | 9536 | 8344 | 54.55 | 45.45 |
| 1 times | 5251 | 4433 | 57.66 | 42.34 | 2506 | 2423 | 56.18 | 43.82 | 2745 | 2010 | 59.38 | 40.62 |
| 2 to 3 times | 4246 | 3514 | 60.39 | 39.61 | 1763 | 1730 | 57.45 | 42.55 | 2483 | 1784 | 63.07 | 36.93 |
| ≥4 | 3537 | 2481 | 65.39 | 34.61 | 1175 | 954 | 57.47 | 42.53 | 2362 | 1527 | 69.14 | 30.86 |

Table D in S1 Text. Multivariate analysis showing association between serious injury occurrence and individual characteristics, including aggressive behaviour indicators (Model 1), with income status (Model 2) and World region (Model 3) added separately

|  |  | | **Model 1** | | | **Model 2** | | | **Model 3** | | |
| --- | --- | --- | --- | --- | --- | --- | --- | --- | --- | --- | --- |
| **Variables** | |  | **OR** | **95% CI** | **p** | **OR** | **95% CI** | **p** | **OR** | **95% CI** | **p** |
| **Demographic characteristics** | |  |  |  |  |  |  |  |  |  |  |
| **Age** | |  |  |  |  |  |  |  |  |  |  |
|  | | ≤13 years (ref) |  |  |  |  |  |  |  |  |  |
|  | | 14 or 15 years | 1.08 | 0.96 - 1.22 | 0.191 | 1.08 | 0.96 - 1.21 | 0.218 | 1.04 | 0.93 - 1.17 | 0.453 |
|  | | ≥ 16 years | 1.05 | 0.93 - 1.19 | 0.429 | 1.03 | 0.91 - 1.16 | 0.673 | 0.95 | 0.85 - 1.07 | 0.418 |
| **Sex** | |  |  |  |  |  |  |  |  |  |  |
|  | | Male (ref) |  |  |  |  |  |  |  |  |  |
|  | | Female | 0.65 | 0.60 - 0.71 | < 0.001 | 0.66 | 0.61 - 0.71 | < 0.001 | 0.67 | 0.62 - 0.73 | < 0.001 |
| **Markers of risky behaviour** | |  |  |  |  |  |  |  |  |  |  |
| **Number of days smoked in the past 30 days** | |  |  |  |  |  |  |  |  |  |  |
|  | | 0 days (ref) |  |  |  |  |  |  |  |  |  |
|  | | 1-5 days | 1.00 | 0.80 - 1.26 | 0.970 | 1.02 | 0.82 - 1.27 | 0.860 | 1.08 | 0.86 - 1.36 | 0.503 |
|  | | 6-29 days | 0.55 | 0.42 - 0.73 | < 0.001 | 0.56 | 0.43 - 0.74 | < 0.001 | 0.59 | 0.44 - 0.79 | < 0.001 |
|  | | All 30 days | 0.63 | 0.44 - 0.89 | 0.009 | 0.64 | 0.45 - 0.90 | 0.012 | 0.72 | 0.51 - 1.01 | 0.059 |
| **Number of days of alcohol in the past 30 days** | |  |  |  |  |  |  |  |  |  |  |
|  | | 0 days (ref) |  |  |  |  |  |  |  |  |  |
|  | | 1-5 days | 1.25 | 1.12 - 1.41 | < 0.001 | 1.23 | 1.10 - 1.38 | < 0.001 | 1.24 | 1.11 - 1.38 | < 0.001 |
|  | | 6-29 days | 0.99 | 0.81 - 1.22 | 0.956 | 0.96 | 0.79 - 1.18 | 0.700 | 0.94 | 0.76 - 1.15 | 0.538 |
|  | | All 30 days | 1.22 | 0.79 - 1.86 | 0.366 | 1.19 | 0.78 - 1.82 | 0.424 | 1.08 | 0.69 - 1.68 | 0.734 |
| **Ever used drugs** | |  |  |  |  |  |  |  |  |  |  |
|  | | No (ref) |  |  |  |  |  |  |  |  |  |
|  | | Yes | 1.93 | 1.59 - 2.35 | < 0.001 | 1.95 | 1.60 - 2.37 | < 0.001 | 1.81 | 1.50 - 2.18 | < 0.001 |
| **Physical activity in the past 7 days** | |  |  |  |  |  |  |  |  |  |  |
|  | | 0 days (ref) |  |  |  |  |  |  |  |  |  |
|  | | 1 day | 1.00 | 0.91 - 1.10 | 0.978 | 0.99 | 0.90 - 1.09 | 0.861 | 1.01 | 0.91 - 1.10 | 0.922 |
|  | | 2 days | 0.95 | 0.84 - 1.08 | 0.429 | 0.93 | 0.82 - 1.05 | 0.264 | 0.93 | 0.83 - 1.05 | 0.255 |
|  | | ≥ 3 days | 1.05 | 0.94 - 1.16 | 0.431 | 1.04 | 0.93 - 1.16 | 0.535 | 1.00 | 0.90 - 1.11 | 0.983 |
| **Contextual factors** | |  |  |  |  |  |  |  |  |  |  |
| **Go hungry in the past 30 days** | |  |  |  |  |  |  |  |  |  |  |
|  | | Never (ref) |  |  |  |  |  |  |  |  |  |
|  | | Rarely | 1.18 | 1.07 - 1.29 | < 0.001 | 1.18 | 1.07 - 1.30 | < 0.001 | 1.23 | 1.12 - 1.35 | < 0.001 |
|  | | Sometimes | 1.27 | 1.15 - 1.41 | < 0.001 | 1.29 | 1.16 - 1.43 | < 0.001 | 1.28 | 1.16 - 1.43 | < 0.001 |
|  | | Most of the time/Always | 1.57 | 1.29 - 1.91 | < 0.001 | 1.57 | 1.29 - 1.91 | < 0.001 | 1.50 | 1.22 - 1.84 | < 0.001 |
| **Number of days bullying in the past 30 days** | |  |  |  |  |  |  |  |  |  |  |
|  | | 0 days (ref) |  |  |  |  |  |  |  |  |  |
|  | | 1-5 days | 2.08 | 1.89 - 2.30 | < 0.001 | 2.07 | 1.88 - 2.28 | < 0.001 | 2.03 | 1.83 - 2.24 | < 0.001 |
|  | | 6-29 days | 2.25 | 1.84 - 2.75 | < 0.001 | 2.26 | 1.85 - 2.76 | < 0.001 | 2.24 | 1.83 - 2.74 | < 0.001 |
|  | | All 30 days | 1.89 | 1.45 - 2.46 | < 0.001 | 1.89 | 1.45 - 2.47 | < 0.001 | 1.89 | 1.45 - 2.46 | < 0.001 |
| **Feeling lonely** | |  |  |  |  |  |  |  |  |  |  |
|  | | Never (ref) |  |  |  |  |  |  |  |  |  |
|  | | Rarely | 1.02 | 0.90 - 1.16 | 0.734 | 1.02 | 0.90 - 1.16 | 0.734 | 1.07 | 0.94 - 1.20 | 0.321 |
|  | | Sometimes | 1.26 | 1.12 - 1.41 | < 0.001 | 1.27 | 1.14 - 1.42 | < 0.001 | 1.30 | 1.16 - 1.45 | < 0.001 |
|  | | Most of the time/Always | 1.40 | 1.21 - 1.62 | < 0.001 | 1.42 | 1.22 - 1.64 | < 0.001 | 1.46 | 1.26 - 1.69 | < 0.001 |
| **Protective factors** | |  |  |  |  |  |  |  |  |  |  |
| **Number of close friends** | |  |  |  |  |  |  |  |  |  |  |
|  | | 0 (ref) |  |  |  |  |  |  |  |  |  |
|  | | 1 | 1.11 | 0.91 - 1.35 | 0.309 | 1.11 | 0.91 - 1.35 | 0.300 | 1.06 | 0.87 - 1.29 | 0.548 |
|  | | 2 | 0.98 | 0.79 - 1.20 | 0.804 | 0.98 | 0.80 - 1.20 | 0.833 | 0.97 | 0.79 - 1.19 | 0.789 |
|  | | 3 or more | 1.01 | 0.87 - 1.18 | 0.873 | 1.03 | 0.88 - 1.19 | 0.731 | 1.17 | 1.01 - 1.37 | 0.043 |
| **Parents or guardians understand problems and worries** | |  |  |  |  |  |  |  |  |  |  |
|  | | Never (ref) |  |  |  |  |  |  |  |  |  |
|  | | Rarely | 1.09 | 0.93 - 1.28 | 0.295 | 1.08 | 0.92 - 1.27 | 0.331 | 1.09 | 0.93 - 1.28 | 0.272 |
|  | | Sometimes | 1.01 | 0.91 - 1.12 | 0.835 | 1.01 | 0.91 - 1.13 | 0.787 | 1.00 | 0.90 - 1.12 | 0.935 |
|  | | Most of the time/Always | 1.18 | 1.06 - 1.31 | 0.003 | 1.17 | 1.05 - 1.30 | 0.004 | 1.13 | 1.02 - 1.27 | 0.025 |
| **Indicators of poor mental health** | |  |  |  |  |  |  |  |  |  |  |
| **Considered or planned suicide** | |  |  |  |  |  |  |  |  |  |  |
|  | | No (ref) |  |  |  |  |  |  |  |  |  |
|  | | Yes | 1.04 | 0.92 - 1.16 | 0.555 | 1.02 | 0.91 - 1.15 | 0.739 | 1.00 | 0.89 - 1.12 | 0.982 |
| **Attempted suicide** | |  |  |  |  |  |  |  |  |  |  |
|  | | No (ref) |  |  |  |  |  |  |  |  |  |
|  | | Yes | 1.69 | 1.46 - 1.97 | < 0.001 | 1.68 | 1.44 - 1.95 | < 0.001 | 1.64 | 1.41 - 1.91 | < 0.001 |
| **Too worried to sleep** | |  |  |  |  |  |  |  |  |  |  |
|  | | Never (ref) |  |  |  |  |  |  |  |  |  |
|  | | Rarely | 1.12 | 1.02 - 1.24 | 0.023 | 1.12 | 1.01 - 1.24 | 0.028 | 1.15 | 1.04 - 1.27 | 0.005 |
|  | | Sometimes | 1.40 | 1.27 - 1.54 | < 0.001 | 1.40 | 1.27 - 1.55 | < 0.001 | 1.40 | 1.27 - 1.55 | < 0.001 |
|  | | Most of the time/Always | 1.61 | 1.38 - 1.89 | < 0.001 | 1.61 | 1.37 - 1.88 | < 0.001 | 1.60 | 1.37 - 1.88 | < 0.001 |
| **Aggressive behaviour indicators** | |  |  |  |  |  |  |  |  |  |  |
| **Number of times in a physical fight** | |  |  |  |  |  |  |  |  |  |  |
|  | | 0 times (ref) |  |  |  |  |  |  |  |  |  |
|  | | 1 times | 1.80 | 1.59 - 2.03 | < 0.001 | 1.80 | 1.59 - 2.03 | < 0.001 | 1.81 | 1.60 - 2.04 | < 0.001 |
|  | | 2 to 3 times | 2.26 | 2.00 - 2.57 | < 0.001 | 2.26 | 2.00 - 2.56 | < 0.001 | 2.28 | 2.02 - 2.57 | < 0.001 |
|  | | ≥4 times | 2.95 | 2.50 - 3.49 | < 0.001 | 2.93 | 2.48 - 3.46 | < 0.001 | 2.94 | 2.50 - 3.47 | < 0.001 |
| **How many times got into fights as result of alcohol** | |  |  |  |  |  |  |  |  |  |  |
|  | | 0 times (ref) |  |  |  |  |  |  |  |  |  |
|  | | 1-2 times | 1.27 | 1.08 - 1.50 | 0.004 | 1.28 | 1.08 - 1.50 | 0.003 | 1.27 | 1.08 - 1.49 | 0.004 |
|  | | ≥3 times | 1.39 | 1.10 - 1.76 | 0.006 | 1.40 | 1.10 - 1.77 | 0.006 | 1.43 | 1.13 - 1.82 | 0.003 |
| **Number of times physically attacked** | |  |  |  |  |  |  |  |  |  |  |
|  | | 0 times (ref) |  |  |  |  |  |  |  |  |  |
|  | | 1 time | 1.61 | 1.41 - 1.84 | < 0.001 | 1.62 | 1.42 - 1.85 | < 0.001 | 1.59 | 1.40 - 1.80 | < 0.001 |
|  | | 2 to 3 times | 1.96 | 1.70 - 2.25 | < 0.001 | 1.98 | 1.72 - 2.27 | < 0.001 | 1.95 | 1.70 - 2.24 | < 0.001 |
|  | | ≥4 times | 3.00 | 2.48 - 3.63 | < 0.001 | 3.05 | 2.52 - 3.68 | < 0.001 | 2.89 | 2.42 - 3.46 | < 0.001 |
|  | |  |  |  |  |  |  |  |  |  |  |
| **Country level factors** | |  |  |  |  |  |  |  |  |  |  |
| **Income Category** | |  |  |  |  |  |  |  |  |  |  |
|  | | Low |  |  |  | 1.72 | 1.41 - 2.09 | < 0.001 |  |  |  |
|  | | Low middle (ref) |  |  |  |  |  |  |  |  |  |
|  | | Upper middle |  |  |  | 1.07 | 0.99 - 1.17 | 0.089 |  |  |  |
|  | | High |  |  |  | 1.14 | 1.01 - 1.28 | 0.033 |  |  |  |
| **World region** | |  |  |  |  |  |  |  |  |  |  |
|  | | East Asia & Pacific (ref) |  |  |  |  |  |  |  |  |  |
|  | | South Asia |  |  |  |  |  |  | 1.31 | 1.03 - 1.66 | 0.027 |
|  | | Latin America and Caribbean |  |  |  |  |  |  | 1.21 | 1.10 - 1.33 | < 0.001 |
|  | | Sub-Saharan Africa |  |  |  |  |  |  | 2.22 | 1.95 - 2.52 | < 0.001 |

Table E in S1 Text. Characteristics of the total population and by occurrence of any serious injury in the last 12 months, in total – Complete cases used in analysis of type of injury. Weights take into account the 2-stage study design and is also adjusted for the non-response rate of participants, see the methods section for a full explanation

|  | **Total** | | **No serious Injuries** | | **≥1 Serious Injury** | |
| --- | --- | --- | --- | --- | --- | --- |
|  | **(Unweighted N)** | **(Weighted %)** | **(Unweighted N)** | **(Weighted %)** | **(Unweighted N)** | **(Weighted %)** |
| **Injured** | **87033** | **100.00** | **56478** | **63.78** | **30555** | **36.22** |
|  |  |  |  |  |  |  |
| **Income status** |  |  |  |  |  |  |
| Low | 2637 | 2.18 | 1360 | 63.96 | 1277 | 36.04 |
| Lower middle | 29962 | 81.94 | 19099 | 51.63 | 10863 | 48.37 |
| Upper middle | 46200 | 15.53 | 30662 | 64.50 | 15538 | 35.50 |
| High | 8234 | 0.35 | 5357 | 64.37 | 2877 | 35.63 |
| **World region** |  |  |  |  |  |  |
| East Asia and Pacific | 45928 | 66.87 | 30626 | 67.78 | 15302 | 32.22 |
| South Asia | 2193 | 15.53 | 1467 | 57.74 | 726 | 42.26 |
| Latin America & Caribbean | 31571 | 9.99 | 20756 | 61.15 | 10815 | 38.85 |
| Sub-Saharan Africa | 7341 | 7.60 | 3629 | 44.39 | 3712 | 55.61 |
| **Demographic characteristics** |  |  |  |  |  |  |
| **Age** |  |  |  |  |  |  |
| ≤13 years | 20891 | 30.88 | 13616 | 65.95 | 7275 | 34.05 |
| 14 or 15 years | 40027 | 45.91 | 25852 | 62.62 | 14175 | 37.38 |
| ≥16 years | 26115 | 23.22 | 17010 | 63.18 | 9105 | 36.82 |
| **Sex** |  |  |  |  |  |  |
| Male | 40781 | 50.68 | 23769 | 57.51 | 17012 | 42.49 |
| Female | 46252 | 49.32 | 32709 | 70.22 | 13543 | 29.78 |
| **Markers of risky behaviour** |  |  |  |  |  |  |
| **Number of days smoked in the past 30 days** |  |  |  |  |  |  |
| Never (0 days) | 76788 | 90.31 | 51294 | 65.07 | 25494 | 34.93 |
| Rarely (1 or 2/3-5 days) | 6136 | 6.30 | 3074 | 47.73 | 3062 | 52.27 |
| Often (6-9/10-19/20-29 days) | 2460 | 2.18 | 1278 | 61.69 | 1182 | 38.31 |
| Always (all 30 days) | 1649 | 1.21 | 832 | 54.57 | 817 | 45.43 |
| **Number of days of alcohol in the past 30 days** |  |  |  |  |  |  |
| Never (0 days) | 67191 | 89.13 | 45293 | 65.73 | 21898 | 34.27 |
| Rarely (1 or 2/3-5 days) | 15665 | 9.42 | 9027 | 48.07 | 6638 | 51.93 |
| Often (6-9/10-19/20-29 days) | 3624 | 1.24 | 1891 | 46.78 | 1733 | 53.22 |
| Always (all 30 days) | 553 | 0.20 | 267 | 41.30 | 286 | 58.70 |
| **Ever used drugs** |  |  |  |  |  |  |
| No | 81316 | 96.23 | 53967 | 64.99 | 27349 | 35.01 |
| Yes | 5717 | 3.77 | 2511 | 32.82 | 3206 | 67.18 |
| **Physical activity in the past 7 days** |  |  |  |  |  |  |
| 0 days | 19413 | 29.64 | 12960 | 65.39 | 6453 | 34.61 |
| 1 day | 17653 | 22.15 | 11877 | 64.86 | 5776 | 35.14 |
| 2 days | 13015 | 11.27 | 8831 | 64.67 | 4184 | 35.33 |
| ≥ 3 days | 36952 | 36.94 | 22810 | 61.57 | 14142 | 38.43 |
| **Contextual factors** |  |  |  |  |  |  |
| **Go hungry in the past 30 days** |  |  |  |  |  |  |
| Never | 43137 | 42.18 | 30558 | 70.01 | 12579 | 29.99 |
| Rarely | 18689 | 18.33 | 11655 | 62.04 | 7034 | 37.96 |
| Sometimes | 20703 | 33.32 | 12074 | 59.40 | 8629 | 40.60 |
| Most of the time/always | 4504 | 6.18 | 2191 | 49.94 | 2313 | 50.06 |
| **Number of days bullying in the past 30 days** |  |  |  |  |  |  |
| Never (0 days) | 65597 | 72.30 | 46831 | 72.04 | 18766 | 27.96 |
| Rarely (1 or 2/3-5 days) | 17180 | 22.98 | 7964 | 43.36 | 9216 | 56.64 |
| Often (6-9/10-19/20-29 days) | 2833 | 3.43 | 1089 | 34.73 | 1744 | 65.27 |
| Always (all 30 days) | 1423 | 1.29 | 594 | 41.83 | 829 | 58.17 |
| **Feeling lonely** |  |  |  |  |  |  |
| Never | 30272 | 34.10 | 21663 | 71.37 | 8609 | 28.63 |
| Rarely | 21802 | 19.42 | 14376 | 67.71 | 7426 | 32.29 |
| Sometimes | 26626 | 37.24 | 16158 | 59.11 | 10468 | 40.89 |
| Most of the time/Always | 8333 | 9.23 | 4281 | 46.28 | 4052 | 53.72 |
| **Protective factors** |  |  |  |  |  |  |
| **Number of close friends** |  |  |  |  |  |  |
| 0 | 4342 | 4.25 | 2651 | 58.99 | 1691 | 41.01 |
| 1 | 8658 | 10.42 | 5374 | 61.40 | 3284 | 38.60 |
| 2 | 11036 | 12.11 | 6938 | 61.19 | 4098 | 38.81 |
| 3 or more | 62997 | 73.22 | 41515 | 64.82 | 21482 | 35.18 |
| **Parents or guardians understand problems and worries** |  |  |  |  |  |  |
| Never | 18304 | 19.33 | 11923 | 64.07 | 6381 | 35.93 |
| Rarely | 14339 | 12.85 | 9020 | 59.75 | 5319 | 40.25 |
| Sometimes | 21109 | 30.42 | 13337 | 64.89 | 7772 | 35.11 |
| Most of the time/Always | 33281 | 37.39 | 22198 | 64.11 | 11083 | 35.89 |
| **Indicators of poor mental health** |  |  |  |  |  |  |
| **Considered or planned suicide** |  |  |  |  |  |  |
| No | 73550 | 88.77 | 49729 | 65.78 | 23821 | 34.22 |
| Yes | 13483 | 11.24 | 6749 | 47.93 | 6734 | 52.07 |
| **Attempted suicide** |  |  |  |  |  |  |
| No | 78177 | 92.42 | 52566 | 65.86 | 25611 | 34.14 |
| Yes | 8856 | 7.58 | 3912 | 38.35 | 4944 | 61.65 |
| **Too worried to sleep** |  |  |  |  |  |  |
| Never | 33892 | 40.06 | 24713 | 71.80 | 9179 | 28.20 |
| Rarely | 24237 | 21.62 | 15770 | 65.02 | 8467 | 34.98 |
| Sometimes | 22743 | 32.23 | 13146 | 56.81 | 9597 | 43.19 |
| Most of the time/Always | 6161 | 6.09 | 2849 | 43.44 | 3312 | 56.56 |
| **Indicators of aggressive behaviour** |  |  |  |  |  |  |
| **Number of times in a physical fight** |  |  |  |  |  |  |
| 0 times | 63275 | 74.57 | 45747 | 71.87 | 17528 | 28.13 |
| 1 times | 11244 | 12.96 | 5789 | 47.46 | 5455 | 52.54 |
| 2 to 3 times | 7247 | 7.50 | 3160 | 36.12 | 4087 | 63.88 |
| ≥4 times | 5267 | 4.97 | 1782 | 26.69 | 3485 | 73.31 |
| **How many times got into fights as result of alcohol** |  |  |  |  |  |  |
| 0 times | 79562 | 95.37 | 52954 | 65.04 | 26608 | 34.96 |
| 1 to 2 times | 5284 | 3.30 | 2637 | 40.85 | 2647 | 59.15 |
| ≥3 times | 2187 | 1.33 | 887 | 30.01 | 1300 | 69.99 |
| **Number of times physically attacked** |  |  |  |  |  |  |
| 0 times | 63682 | 64.73 | 45927 | 73.53 | 17755 | 26.47 |
| 1 times | 9643 | 13.35 | 4929 | 53.70 | 4714 | 46.30 |
| 2 to 3 times | 7729 | 12.60 | 3493 | 47.68 | 4236 | 52.32 |
| ≥4 times | 5979 | 9.31 | 2129 | 32.21 | 3850 | 67.79 |

Table F in S1 Text. Characteristics of the total population and by occurrence of any serious injury in the last 12 months, by sex – Complete cases used in analysis of type of injury. Weights take into account the 2-stage study design and is also adjusted for the non-response rate of participants, see the methods section for a full explanation

|  | **Total** | | | | **No serious Injuries** | | | | **>1 Serious Injury** | | | |
| --- | --- | --- | --- | --- | --- | --- | --- | --- | --- | --- | --- | --- |
|  | **(Unweighted N)** | **(Unweighted N)** | **(Weighted %)** | **(Weighted %)** | **(Unweighted N)** | **(Unweighted N)** | **(Weighted %)** | **(Weighted %)** | **(Unweighted N)** | **(Unweighted N)** | **(Weighted %)** | **(Weighted %)** |
|  | **Male** | **Female** | **Male** | **Female** | **Male** | **Female** | **Male** | **Female** | **Male** | **Female** | **Male** | **Female** |
| **Injured** | **40781** | **46252** | **50.68** | **49.32** | **23769** | **32709** | **45.69** | **54.31** | **17012** | **13543** | **59.45** | **40.55** |
|  |  |  |  |  |  |  |  |  |  |  |  |  |
| **Income Status** |  |  |  |  |  |  |  |  |  |  |  |  |
| Low | 1643 | 994 | 57.79 | 42.21 | 807 | 553 | 55.62 | 44.38 | 836 | 441 | 60.10 | 39.90 |
| Lower middle | 13580 | 16382 | 50.89 | 49.11 | 7853 | 11246 | 45.94 | 54.06 | 5727 | 5136 | 59.69 | 40.32 |
| Upper middle | 21765 | 24435 | 48.58 | 51.42 | 12893 | 17769 | 43.33 | 56.67 | 8872 | 6666 | 58.12 | 41.88 |
| High | 3793 | 4441 | 48.00 | 52.00 | 2216 | 3141 | 43.59 | 56.41 | 1577 | 1300 | 55.98 | 44.02 |
| **World region** |  |  |  |  |  |  |  |  |  |  |  |  |
| East Asia and Pacific | 20914 | 25014 | 46.68 | 53.32 | 12589 | 18037 | 42.03 | 57.97 | 8325 | 6977 | 56.45 | 43.55 |
| South Asia | 913 | 1280 | 66.60 | 33.40 | 583 | 884 | 62.34 | 37.66 | 330 | 396 | 72.43 | 27.57 |
| Latin America & Caribbean | 14884 | 16687 | 49.32 | 50.68 | 8683 | 12073 | 43.53 | 56.47 | 6201 | 4614 | 58.43 | 41.57 |
| Sub-Saharan Africa | 4070 | 3271 | 55.09 | 44.91 | 1914 | 1715 | 54.61 | 45.39 | 2156 | 1556 | 55.47 | 44.53 |
| **Demographic characteristics** |  |  |  |  |  |  |  |  |  |  |  |  |
| **Age** |  |  |  |  |  |  |  |  |  |  |  |  |
| ≤13 years | 9437 | 11454 | 47.39 | 52.61 | 5424 | 8192 | 41.00 | 59.00 | 4013 | 3262 | 59.77 | 40.23 |
| 14 or 15 years | 18549 | 21478 | 51.97 | 48.03 | 10768 | 15084 | 47.80 | 52.20 | 7781 | 6394 | 58.96 | 41.04 |
| ≥16 years | 12795 | 13320 | 52.48 | 47.52 | 7577 | 9433 | 48.09 | 51.91 | 5218 | 3887 | 60.03 | 39.97 |
| **Markers of risky behaviour** |  |  |  |  |  |  |  |  |  |  |  |  |
| **Number of days smoked in the past 30 days** |  |  |  |  |  |  |  |  |  |  |  |  |
| Never (0 days) | 34203 | 42585 | 47.31 | 52.69 | 20690 | 30604 | 42.48 | 57.52 | 13513 | 11981 | 56.32 | 43.68 |
| Rarely (1 or 2/3-5 days) | 3791 | 2345 | 79.46 | 20.54 | 1713 | 1361 | 79.60 | 20.40 | 2078 | 984 | 79.33 | 20.67 |
| Often (6-9/10-19/20-29 days) | 1582 | 878 | 84.78 | 15.22 | 771 | 507 | 86.78 | 13.22 | 811 | 371 | 81.56 | 18.44 |
| Always (all 30 days) | 1205 | 444 | 90.36 | 9.64 | 595 | 237 | 93.80 | 6.20 | 610 | 207 | 86.23 | 13.77 |
| **Number of days of alcohol in the past 30 days** |  |  |  |  |  |  |  |  |  |  |  |  |
| Never (0 days) | 30710 | 36481 | 49.69 | 50.31 | 18797 | 26496 | 45.09 | 54.91 | 11913 | 9985 | 58.52 | 41.48 |
| Rarely (1 or 2/3-5 days) | 7818 | 7847 | 57.65 | 42.35 | 3964 | 5063 | 51.76 | 48.24 | 3854 | 2784 | 63.11 | 36.89 |
| Often (6-9/10-19/20-29 days) | 1909 | 1715 | 64.79 | 35.21 | 856 | 1035 | 56.72 | 43.28 | 1053 | 680 | 71.88 | 28.12 |
| Always (all 30 days) | 344 | 209 | 72.62 | 27.38 | 152 | 115 | 65.80 | 34.20 | 192 | 94 | 77.43 | 22.57 |
| **Ever used drugs** |  |  |  |  |  |  |  |  |  |  |  |  |
| No | 37303 | 44013 | 49.99 | 50.01 | 22346 | 31621 | 45.25 | 54.75 | 14957 | 12392 | 58.79 | 41.21 |
| Yes | 3478 | 2239 | 68.23 | 31.77 | 1423 | 1088 | 68.36 | 31.64 | 2055 | 1151 | 68.17 | 31.83 |
| **Physical activity in the past 7 days** |  |  |  |  |  |  |  |  |  |  |  |  |
| 0 days | 8235 | 11178 | 47.40 | 52.60 | 4912 | 8048 | 42.76 | 57.24 | 3323 | 3130 | 56.17 | 43.83 |
| 1 day | 7209 | 10444 | 44.23 | 55.77 | 4315 | 7562 | 38.81 | 61.19 | 2894 | 2882 | 54.23 | 45.77 |
| 2 days | 5394 | 7621 | 46.68 | 53.32 | 3322 | 5509 | 43.11 | 56.89 | 2072 | 2112 | 53.23 | 46.77 |
| ≥ 3 days | 19943 | 17009 | 58.38 | 41.62 | 11220 | 11590 | 53.37 | 46.63 | 8723 | 5419 | 66.42 | 33.58 |
| **Contextual factors** |  |  |  |  |  |  |  |  |  |  |  |  |
| **Go hungry in the past 30 days** |  |  |  |  |  |  |  |  |  |  |  |  |
| Never | 19568 | 23569 | 48.66 | 51.34 | 12637 | 17921 | 44.56 | 55.44 | 6931 | 5648 | 58.24 | 41.76 |
| Rarely | 9333 | 9356 | 53.41 | 46.59 | 5245 | 6410 | 49.22 | 50.78 | 4088 | 2946 | 60.27 | 39.73 |
| Sometimes | 9636 | 11067 | 50.87 | 49.13 | 4917 | 7157 | 44.81 | 55.19 | 4719 | 3910 | 59.74 | 40.26 |
| Most of the time/always | 2244 | 2260 | 55.25 | 44.75 | 970 | 1221 | 49.22 | 50.78 | 1274 | 1039 | 61.27 | 38.73 |
| **Number of days bullying in the past 30 days** |  |  |  |  |  |  |  |  |  |  |  |  |
| Never (0 days) | 30168 | 35429 | 49.15 | 50.85 | 19510 | 27321 | 44.74 | 55.26 | 10658 | 8108 | 60.52 | 39.48 |
| Rarely (1 or 2/3-5 days) | 8448 | 8732 | 54.17 | 45.83 | 3486 | 4478 | 49.70 | 50.30 | 4962 | 4254 | 57.59 | 42.41 |
| Often (6-9/10-19/20-29 days) | 1465 | 1368 | 58.82 | 41.18 | 512 | 577 | 53.12 | 46.88 | 953 | 791 | 61.85 | 38.15 |
| Always (all 30 days) | 700 | 723 | 52.20 | 47.80 | 261 | 333 | 47.45 | 52.55 | 439 | 390 | 55.62 | 44.38 |
| **Feeling lonely** |  |  |  |  |  |  |  |  |  |  |  |  |
| Never | 16661 | 13611 | 57.71 | 42.29 | 10944 | 10719 | 52.65 | 47.35 | 5717 | 2892 | 70.34 | 29.66 |
| Rarely | 10615 | 11187 | 53.40 | 46.60 | 6139 | 8237 | 48.67 | 51.33 | 4476 | 2950 | 63.31 | 36.69 |
| Sometimes | 10512 | 16114 | 44.56 | 55.44 | 5408 | 10750 | 37.92 | 62.08 | 5104 | 5364 | 54.16 | 45.84 |
| Most of the time/Always | 2993 | 5340 | 43.63 | 56.37 | 1278 | 3003 | 37.01 | 62.99 | 1715 | 2337 | 49.34 | 50.66 |
| **Protective factors** |  |  |  |  |  |  |  |  |  |  |  |  |
| **Number of close friends** |  |  |  |  |  |  |  |  |  |  |  |  |
| 0 | 2031 | 2311 | 50.38 | 49.62 | 1108 | 1543 | 46.60 | 53.40 | 923 | 768 | 55.83 | 44.17 |
| 1 | 3656 | 5002 | 50.33 | 49.67 | 2113 | 3261 | 49.92 | 50.08 | 1543 | 1741 | 50.98 | 49.02 |
| 2 | 4647 | 6389 | 52.77 | 47.23 | 2591 | 4347 | 49.29 | 50.71 | 2056 | 2042 | 58.27 | 41.73 |
| 3 or more | 30447 | 32550 | 50.40 | 49.60 | 17957 | 23558 | 44.52 | 55.48 | 12490 | 8992 | 61.23 | 38.77 |
| **Parents or guardians understand problems and worries** |  |  |  |  |  |  |  |  |  |  |  |  |
| Never | 8977 | 9327 | 55.46 | 44.54 | 5368 | 6555 | 50.73 | 49.27 | 3609 | 2772 | 63.88 | 36.12 |
| Rarely | 6599 | 7740 | 50.45 | 49.55 | 3687 | 5333 | 46.05 | 53.95 | 2912 | 2407 | 56.97 | 43.03 |
| Sometimes | 9879 | 11230 | 49.50 | 50.50 | 5555 | 7782 | 44.42 | 55.58 | 4324 | 3448 | 58.89 | 41.11 |
| Most of the time/Always | 15326 | 17955 | 49.24 | 50.76 | 9159 | 13039 | 44.03 | 55.97 | 6167 | 4916 | 58.54 | 41.46 |
| **Indicators of mental health** |  |  |  |  |  |  |  |  |  |  |  |  |
| **Considered or planned suicide** |  |  |  |  |  |  |  |  |  |  |  |  |
| No | 35565 | 37985 | 51.46 | 48.54 | 21589 | 28140 | 46.31 | 53.69 | 13976 | 9845 | 61.37 | 38.63 |
| Yes | 5216 | 8267 | 44.47 | 55.53 | 2180 | 4569 | 39.02 | 60.98 | 3036 | 3698 | 49.48 | 50.52 |
| **Attempted suicide** |  |  |  |  |  |  |  |  |  |  |  |  |
| No | 37205 | 40972 | 51.06 | 48.94 | 22457 | 30109 | 45.98 | 54.02 | 14748 | 10863 | 60.87 | 39.13 |
| Yes | 3576 | 5280 | 45.97 | 54.03 | 1312 | 2600 | 39.73 | 60.27 | 2264 | 2680 | 49.85 | 50.15 |
| **Too worried to sleep** |  |  |  |  |  |  |  |  |  |  |  |  |
| Never | 18302 | 15590 | 55.64 | 44.36 | 12301 | 12412 | 50.72 | 49.28 | 6001 | 3178 | 68.15 | 31.85 |
| Rarely | 11064 | 13173 | 50.05 | 49.95 | 6178 | 9592 | 45.28 | 54.72 | 4886 | 3581 | 58.93 | 41.07 |
| Sometimes | 9111 | 13632 | 46.13 | 53.87 | 4419 | 8727 | 39.23 | 60.77 | 4692 | 4905 | 55.20 | 44.80 |
| Most of the time/Always | 2304 | 3857 | 44.32 | 55.68 | 871 | 1978 | 37.95 | 62.05 | 1433 | 1879 | 49.21 | 50.79 |
| **Indicators of aggressive behaviour** |  |  |  |  |  |  |  |  |  |  |  |  |
| **Number of times in a physical fight** |  |  |  |  |  |  |  |  |  |  |  |  |
| 0 times | 25986 | 37289 | 45.31 | 54.69 | 17306 | 28441 | 41.79 | 58.21 | 8680 | 8848 | 54.31 | 45.69 |
| 1 times | 6661 | 4583 | 63.87 | 36.13 | 3332 | 2457 | 64.72 | 35.28 | 3329 | 2126 | 63.10 | 36.90 |
| 2 to 3 times | 4575 | 2672 | 66.82 | 33.18 | 1957 | 1203 | 67.74 | 32.26 | 2618 | 1469 | 66.30 | 33.70 |
| ≥4 times | 3559 | 1708 | 72.38 | 27.62 | 1174 | 608 | 70.24 | 29.76 | 2385 | 1100 | 73.16 | 26.84 |
| **How many times got into fights as result of alcohol** |  |  |  |  |  |  |  |  |  |  |  |  |
| 0 times | 36850 | 42712 | 50.19 | 49.81 | 22146 | 30808 | 45.37 | 54.63 | 14704 | 11904 | 59.14 | 40.86 |
| 1 to 2 times | 2733 | 2551 | 59.70 | 40.30 | 1186 | 1451 | 56.20 | 43.80 | 1547 | 1100 | 62.12 | 37.88 |
| >3 times | 1198 | 989 | 63.48 | 36.52 | 437 | 450 | 60.12 | 39.88 | 761 | 539 | 64.92 | 35.08 |
| **Number of times physically attacked** |  |  |  |  |  |  |  |  |  |  |  |  |
| 0 times | 27808 | 35874 | 45.25 | 54.75 | 18325 | 27602 | 41.89 | 58.11 | 9483 | 8272 | 54.57 | 45.43 |
| 1 times | 5227 | 4416 | 57.59 | 42.41 | 2506 | 2423 | 56.18 | 43.82 | 2721 | 1993 | 59.23 | 40.77 |
| 2 to 3 times | 4232 | 3497 | 60.42 | 39.58 | 1763 | 1730 | 57.45 | 42.55 | 2469 | 1767 | 63.12 | 36.88 |
| ≥4 | 3514 | 2465 | 65.32 | 34.68 | 1175 | 954 | 57.47 | 42.53 | 2339 | 1511 | 69.05 | 30.95 |

Table G in S1 Text. Characteristics of the total population and by occurrence of any serious injury in the last 12 months, in total - Complete cases used in analysis of mechanism of injury. Weights take into account the 2-stage study design and is also adjusted for the non-response rate of participants, see the methods section for a full explanation. *Data on mechanism of injury were not captured in the Bangladesh survey.

|  | **Total** | | **No serious Injuries** | | **≥1 Serious Injury** | |
| --- | --- | --- | --- | --- | --- | --- |
|  | **(Unweighted N)** | **(Weighted %)** | **(Unweighted N)** | **(Weighted %)** | **(Unweighted N)** | **(Weighted %)** |
| **Injured** | **84883** | **100.00** | **55011** | **64.86** | **29872** | **35.14** |
|  |  |  |  |  |  |  |
| **Income Status** |  |  |  |  |  |  |
| Low | 2638 | 2.60 | 1360 | 65.40 | 1278 | 34.60 |
| Lower middle | 27772 | 78.61 | 17632 | 51.37 | 10140 | 48.63 |
| Upper middle | 46234 | 18.38 | 30662 | 64.49 | 15572 | 35.51 |
| High | 8239 | 0.41 | 5357 | 64.53 | 2882 | 35.47 |
| **World region*** |  |  |  |  |  |  |
| East Asia and Pacific | 45928 | 79.16 | 30626 | 67.76 | 15302 | 32.24 |
| Latin America & Caribbean | 31607 | 11.83 | 20756 | 61.14 | 10851 | 38.86 |
| Sub-Saharan Africa | 7348 | 9.01 | 3629 | 44.31 | 3719 | 55.69 |
| **Demographic characteristics** |  |  |  |  |  |  |
| **Age** |  |  |  |  |  |  |
| ≤13 years | 20394 | 31.78 | 13285 | 68.06 | 7109 | 31.94 |
| 14 or 15 years | 38513 | 42.43 | 24810 | 63.25 | 13703 | 36.75 |
| ≥16 years | 25976 | 25.80 | 16916 | 63.57 | 9060 | 36.43 |
| **Sex** |  |  |  |  |  |  |
| Male | 39875 | 47.75 | 23186 | 58.37 | 16689 | 41.63 |
| Female | 45008 | 52.25 | 31825 | 70.80 | 13183 | 29.20 |
| **Markers of risky behaviour** |  |  |  |  |  |  |
| **Number of days smoked in the past 30 days** |  |  |  |  |  |  |
| Never (0 days) | 74789 | 90.11 | 49977 | 66.69 | 24812 | 33.31 |
| Rarely (1 or 2/3-5 days) | 6106 | 6.66 | 3047 | 45.95 | 3059 | 54.05 |
| Often (6-9/10-19/20-29 days) | 2333 | 1.85 | 1156 | 50.70 | 1177 | 49.30 |
| Always (all 30 days) | 1655 | 1.38 | 831 | 56.00 | 824 | 44.00 |
| **Number of days of alcohol in the past 30 days** |  |  |  |  |  |  |
| Never (0 days) | 65032 | 87.34 | 43831 | 67.29 | 21201 | 32.71 |
| Rarely (1 or 2/3-5 days) | 15675 | 11.02 | 9025 | 48.43 | 6650 | 51.57 |
| Often (6-9/10-19/20-29 days) | 3624 | 1.39 | 1888 | 46.72 | 1736 | 53.28 |
| Always (all 30 days) | 552 | 0.25 | 267 | 40.63 | 285 | 59.37 |
| **Ever used drugs** |  |  |  |  |  |  |
| No | 79175 | 95.91 | 52505 | 66.17 | 26670 | 33.83 |
| Yes | 5708 | 4.09 | 2506 | 34.29 | 3202 | 65.71 |
| **Physical activity in the past 7 days** |  |  |  |  |  |  |
| 0 days | 18965 | 31.06 | 12666 | 66.45 | 6299 | 33.55 |
| 1 day | 17527 | 24.92 | 11831 | 66.48 | 5696 | 33.52 |
| 2 days | 12968 | 12.76 | 8803 | 65.46 | 4165 | 34.54 |
| ≥ 3 days | 35423 | 31.28 | 21711 | 61.76 | 13712 | 38.24 |
| **Contextual factors** |  |  |  |  |  |  |
| **Go hungry in the past 30 days** |  |  |  |  |  |  |
| Never | 42370 | 42.79 | 30074 | 72.06 | 12296 | 27.94 |
| Rarely | 18524 | 20.16 | 11520 | 61.84 | 7004 | 38.16 |
| Sometimes | 19780 | 32.00 | 11419 | 59.82 | 8361 | 40.18 |
| Most of the time/always | 4209 | 5.05 | 1998 | 47.93 | 2211 | 52.07 |
| **Number of days bullying in the past 30 days** |  |  |  |  |  |  |
| Never (0 days) | 63853 | 71.43 | 45535 | 73.41 | 18318 | 26.59 |
| Rarely (1 or 2/3-5 days) | 16811 | 23.46 | 7803 | 44.61 | 9008 | 55.39 |
| Often (6-9/10-19/20-29 days) | 2797 | 3.61 | 1079 | 36.61 | 1718 | 63.39 |
| Always (all 30 days) | 1422 | 1.50 | 594 | 42.45 | 828 | 57.55 |
| **Feeling lonely** |  |  |  |  |  |  |
| Never | 29435 | 32.12 | 21102 | 73.95 | 8333 | 26.05 |
| Rarely | 21329 | 20.52 | 13920 | 65.19 | 7409 | 34.81 |
| Sometimes | 25939 | 38.14 | 15774 | 60.98 | 10165 | 39.02 |
| Most of the time/Always | 8180 | 9.22 | 4215 | 48.56 | 3965 | 51.44 |
| **Protective factors** |  |  |  |  |  |  |
| **Number of close friends** |  |  |  |  |  |  |
| 0 | 4176 | 3.69 | 2543 | 59.82 | 1633 | 40.18 |
| 1 | 7929 | 7.75 | 4827 | 56.85 | 3102 | 43.15 |
| 2 | 10502 | 9.38 | 6552 | 58.93 | 3950 | 41.07 |
| 3 or more | 62276 | 79.18 | 41089 | 66.59 | 21187 | 33.41 |
| **Parents or guardians understand problems and worries** |  |  |  |  |  |  |
| Never | 18102 | 20.50 | 11808 | 66.63 | 6294 | 33.37 |
| Rarely | 14223 | 13.70 | 8961 | 60.04 | 5262 | 39.96 |
| Sometimes | 20304 | 30.32 | 12704 | 64.23 | 7600 | 35.77 |
| Most of the time/Always | 32254 | 35.48 | 21538 | 66.24 | 10716 | 33.76 |
| **Indicators of mental health** |  |  |  |  |  |  |
| **Considered or planned suicide** |  |  |  |  |  |  |
| No | 71535 | 88.21 | 48346 | 67.11 | 23189 | 32.89 |
| Yes | 13348 | 11.80 | 6665 | 48.09 | 6683 | 51.91 |
| **Attempted suicide** |  |  |  |  |  |  |
| No | 76098 | 91.94 | 51135 | 67.08 | 24963 | 32.92 |
| Yes | 8785 | 8.06 | 3876 | 39.52 | 4909 | 60.48 |
| **Too worried to sleep** |  |  |  |  |  |  |
| Never | 33025 | 39.04 | 24102 | 73.97 | 8923 | 26.03 |
| Rarely | 23706 | 22.53 | 15298 | 62.77 | 8408 | 37.23 |
| Sometimes | 22063 | 31.89 | 12798 | 59.48 | 9265 | 40.52 |
| Most of the time/Always | 6089 | 6.54 | 2813 | 43.96 | 3276 | 56.04 |
| **Indicators of aggressive behaviour** |  |  |  |  |  |  |
| **Number of times in a physical fight** |  |  |  |  |  |  |
| 0 times | 61420 | 73.81 | 44388 | 73.00 | 17032 | 27.00 |
| 1 times | 11109 | 13.65 | 5725 | 48.48 | 5384 | 51.52 |
| 2 to 3 times | 7157 | 7.72 | 3132 | 38.16 | 4025 | 61.84 |
| ≥4 times | 5197 | 4.82 | 1766 | 29.45 | 3431 | 70.55 |
| **How many times got into fights as result of alcohol** |  |  |  |  |  |  |
| 0 times | 77410 | 94.66 | 51488 | 66.37 | 25922 | 33.63 |
| 1 to 2 times | 5291 | 3.87 | 2636 | 40.43 | 2655 | 59.57 |
| >3 times | 2182 | 1.47 | 887 | 32.20 | 1295 | 67.80 |
| **Number of times physically attacked** |  |  |  |  |  |  |
| 0 times | 62678 | 69.62 | 45029 | 72.90 | 17649 | 27.10 |
| 1 times | 9356 | 13.04 | 4736 | 51.17 | 4620 | 48.83 |
| 2 to 3 times | 7297 | 10.98 | 3264 | 47.17 | 4033 | 52.83 |
| ≥4 | 5552 | 6.36 | 1982 | 35.52 | 3570 | 64.48 |

Table H in S1 Text. Characteristics of the total population and by occurrence of any serious injury in the last 12 months by sex - Complete cases used in analysis of mechanism of injury. Weights take into account the 2-stage study design and is also adjusted for the non-response rate of participants, see the methods section for a full explanation. *Data on mechanism of injury were not captured in the Bangladesh survey.

|  | | | | | | | | | | | | |
| --- | --- | --- | --- | --- | --- | --- | --- | --- | --- | --- | --- | --- |
|  | **Total** | | | | **No serious Injuries** | | | | **≥1 Serious Injury** | | | |
|  | **(Unweighted N)** | **(Unweighted N)** | **(Weighted %)** | **(Weighted %)** | **(Unweighted N)** | **(Unweighted N)** | **(Weighted %)** | **(Weighted %)** | **(Unweighted N)** | **(Unweighted N)** | **(Weighted %)** | **(Weighted %)** |
|  | **Male** | **Female** | **Male** | **Female** | **Male** | **Female** | **Male** | **Female** | **Male** | **Female** | **Male** | **Female** |
| **Injured** | **39875** | **45008** | **47.75** | **52.25** | **23186** | **31825** | **42.97** | **57.03** | **16689** | **13183** | **56.58** | **43.42** |
|  |  |  |  |  |  |  |  |  |  |  |  |  |
| **Income Status** |  |  |  |  |  |  |  |  |  |  |  |  |
| Low | 1642 | 996 | 57.61 | 42.39 | 807 | 553 | 55.62 | 44.38 | 835 | 443 | 59.71 | 40.29 |
| Lower middle | 12667 | 15105 | 47.24 | 52.76 | 7270 | 10362 | 42.56 | 57.44 | 5397 | 4743 | 56.08 | 43.92 |
| Upper middle | 21774 | 24460 | 48.55 | 51.45 | 12893 | 17769 | 43.33 | 56.67 | 8881 | 6691 | 58.04 | 41.96 |
| High | 3792 | 4447 | 47.97 | 52.03 | 2216 | 3141 | 43.59 | 56.41 | 1576 | 1306 | 55.93 | 44.07 |
| **World region*** |  |  |  |  |  |  |  |  |  |  |  |  |
| East Asia and Pacific | 20912 | 25016 | 46.70 | 53.30 | 12589 | 18037 | 42.03 | 57.97 | 8323 | 6979 | 56.50 | 43.50 |
| Latin America & Caribbean | 14892 | 16715 | 49.26 | 50.74 | 8683 | 12073 | 43.53 | 56.47 | 6209 | 4642 | 58.27 | 41.73 |
| Sub-Saharan Africa | 4071 | 3277 | 55.04 | 44.96 | 1914 | 1715 | 54.61 | 45.39 | 2157 | 1562 | 55.39 | 44.61 |
| **Demographic characteristics** |  |  |  |  |  |  |  |  |  |  |  |  |
| **Age** |  |  |  |  |  |  |  |  |  |  |  |  |
| ≤13 years | 9290 | 11104 | 45.22 | 54.78 | 5362 | 7923 | 40.40 | 59.60 | 3928 | 3181 | 55.49 | 44.51 |
| 14 or 15 years | 17885 | 20628 | 48.17 | 51.83 | 10310 | 14500 | 43.31 | 56.69 | 7575 | 6128 | 56.56 | 43.44 |
| ≥16 years | 12700 | 13276 | 50.17 | 49.83 | 7514 | 9402 | 45.81 | 54.19 | 5186 | 3874 | 57.78 | 42.22 |
| **Markers of risky behaviour** |  |  |  |  |  |  |  |  |  |  |  |  |
| **Number of days smoked in the past 30 days** |  |  |  |  |  |  |  |  |  |  |  |  |
| Never (0 days) | 33444 | 41345 | 44.23 | 55.77 | 20244 | 29733 | 40.02 | 59.98 | 13200 | 11612 | 52.64 | 47.36 |
| Rarely (1 or 2/3-5 days) | 3755 | 2351 | 77.54 | 22.46 | 1687 | 1360 | 76.59 | 23.41 | 2068 | 991 | 78.35 | 21.65 |
| Often (6-9/10-19/20-29 days) | 1467 | 866 | 80.65 | 19.35 | 661 | 495 | 80.29 | 19.71 | 806 | 371 | 81.02 | 18.98 |
| Always (all 30 days) | 1209 | 446 | 90.03 | 9.97 | 594 | 237 | 93.76 | 6.24 | 615 | 209 | 85.29 | 14.71 |
| **Number of days of alcohol in the past 30 days** |  |  |  |  |  |  |  |  |  |  |  |  |
| Never (0 days) | 29807 | 35225 | 46.25 | 53.75 | 18219 | 25612 | 42.03 | 57.97 | 11588 | 9613 | 54.94 | 45.06 |
| Rarely (1 or 2/3-5 days) | 7820 | 7855 | 57.18 | 42.82 | 3962 | 5063 | 51.54 | 48.46 | 3858 | 2792 | 62.47 | 37.53 |
| Often (6-9/10-19/20-29 days) | 1904 | 1720 | 62.62 | 37.38 | 853 | 1035 | 54.18 | 45.82 | 1051 | 685 | 70.03 | 29.97 |
| Always (all 30 days) | 344 | 208 | 73.53 | 26.47 | 152 | 115 | 65.80 | 34.20 | 192 | 93 | 78.82 | 21.18 |
| **Ever used drugs** |  |  |  |  |  |  |  |  |  |  |  |  |
| No | 36407 | 42768 | 46.95 | 53.05 | 21768 | 30737 | 42.44 | 57.56 | 14639 | 12031 | 55.78 | 44.22 |
| Yes | 3468 | 2240 | 66.46 | 33.54 | 1418 | 1088 | 66.97 | 33.03 | 2050 | 1152 | 66.19 | 33.81 |
| **Physical activity in the past 7 days** |  |  |  |  |  |  |  |  |  |  |  |  |
| 0 days | 8098 | 10867 | 46.41 | 53.59 | 4843 | 7823 | 42.07 | 57.93 | 3255 | 3044 | 55.02 | 44.98 |
| 1 day | 7148 | 10379 | 42.80 | 57.20 | 4301 | 7530 | 38.19 | 61.81 | 2847 | 2849 | 51.95 | 48.05 |
| 2 days | 5376 | 7592 | 45.81 | 54.19 | 3309 | 5494 | 42.31 | 57.69 | 2067 | 2098 | 52.43 | 47.57 |
| ≥ 3 days | 19253 | 16170 | 53.81 | 46.19 | 10733 | 10978 | 48.32 | 51.68 | 8520 | 5192 | 62.69 | 37.31 |
| **Contextual factors** |  |  |  |  |  |  |  |  |  |  |  |  |
| **Go hungry in the past 30 days** |  |  |  |  |  |  |  |  |  |  |  |  |
| Never | 19272 | 23098 | 45.66 | 54.34 | 12470 | 17604 | 41.88 | 58.12 | 6802 | 5494 | 55.42 | 44.58 |
| Rarely | 9237 | 9287 | 51.56 | 48.44 | 5172 | 6348 | 47.33 | 52.67 | 4065 | 2939 | 58.41 | 41.59 |
| Sometimes | 9248 | 10532 | 47.37 | 52.63 | 4662 | 6757 | 41.67 | 58.33 | 4586 | 3775 | 55.86 | 44.14 |
| Most of the time/always | 2118 | 2091 | 52.65 | 47.35 | 882 | 1116 | 44.71 | 55.29 | 1236 | 975 | 59.96 | 40.04 |
| **Number of days bullying in the past 30 days** |  |  |  |  |  |  |  |  |  |  |  |  |
| Never (0 days) | 29468 | 34385 | 46.28 | 53.72 | 19009 | 26526 | 41.98 | 58.02 | 10459 | 7859 | 58.15 | 41.85 |
| Rarely (1 or 2/3-5 days) | 8264 | 8547 | 50.56 | 49.44 | 3408 | 4395 | 46.57 | 53.43 | 4856 | 4152 | 53.77 | 46.23 |
| Often (6-9/10-19/20-29 days) | 1442 | 1355 | 56.43 | 43.57 | 508 | 571 | 51.60 | 48.40 | 934 | 784 | 59.21 | 40.79 |
| Always (all 30 days) | 701 | 721 | 53.05 | 46.95 | 261 | 333 | 47.45 | 52.55 | 440 | 388 | 57.18 | 42.82 |
| **Feeling lonely** |  |  |  |  |  |  |  |  |  |  |  |  |
| Never | 16344 | 13091 | 55.27 | 44.73 | 10757 | 10345 | 50.36 | 49.64 | 5587 | 2746 | 69.20 | 30.80 |
| Rarely | 10356 | 10973 | 51.83 | 48.17 | 5894 | 8026 | 45.86 | 54.14 | 4462 | 2947 | 63.01 | 36.99 |
| Sometimes | 10251 | 15688 | 41.45 | 58.55 | 5281 | 10493 | 35.42 | 64.58 | 4970 | 5195 | 50.86 | 49.14 |
| Most of the time/Always | 2924 | 5256 | 38.55 | 61.45 | 1254 | 2961 | 34.33 | 65.67 | 1670 | 2295 | 42.54 | 57.46 |
| **Protective factors** |  |  |  |  |  |  |  |  |  |  |  |  |
| **Number of close friends** |  |  |  |  |  |  |  |  |  |  |  |  |
| 0 | 1993 | 2183 | 51.64 | 48.36 | 1087 | 1456 | 49.11 | 50.89 | 906 | 727 | 55.42 | 44.58 |
| 1 | 3335 | 4594 | 43.63 | 56.37 | 1856 | 2971 | 40.85 | 59.15 | 1479 | 1623 | 47.29 | 52.71 |
| 2 | 4440 | 6062 | 44.73 | 55.27 | 2457 | 4095 | 40.13 | 59.87 | 1983 | 1967 | 51.32 | 48.68 |
| 3 or more | 30107 | 32169 | 48.33 | 51.67 | 17786 | 23303 | 43.19 | 56.81 | 12321 | 8866 | 58.58 | 41.42 |
| **Parents or guardians understand problems and worries** |  |  |  |  |  |  |  |  |  |  |  |  |
| Never | 8880 | 9222 | 53.43 | 46.57 | 5326 | 6482 | 49.88 | 50.12 | 3554 | 2740 | 60.53 | 39.47 |
| Rarely | 6531 | 7692 | 47.29 | 52.71 | 3645 | 5316 | 41.81 | 58.19 | 2886 | 2376 | 55.54 | 44.46 |
| Sometimes | 9512 | 10792 | 46.34 | 53.66 | 5276 | 7428 | 41.00 | 59.00 | 4236 | 3364 | 55.94 | 44.06 |
| Most of the time/Always | 14952 | 17302 | 45.85 | 54.15 | 8939 | 12599 | 41.00 | 59.00 | 6013 | 4703 | 55.36 | 44.64 |
| **Indicators of mental health** |  |  |  |  |  |  |  |  |  |  |  |  |
| **Considered or planned suicide** |  |  |  |  |  |  |  |  |  |  |  |  |
| No | 34706 | 36829 | 48.48 | 51.52 | 21028 | 27318 | 43.54 | 56.46 | 13678 | 9511 | 58.55 | 41.45 |
| Yes | 5169 | 8179 | 42.32 | 57.68 | 2158 | 4507 | 37.02 | 62.98 | 3011 | 3672 | 47.23 | 52.77 |
| **Attempted suicide** |  |  |  |  |  |  |  |  |  |  |  |  |
| No | 36334 | 39764 | 48.11 | 51.89 | 21887 | 29248 | 43.17 | 56.83 | 14447 | 10516 | 58.19 | 41.81 |
| Yes | 3541 | 5244 | 43.62 | 56.38 | 1299 | 2577 | 39.11 | 60.89 | 2242 | 2667 | 46.57 | 53.43 |
| **Too worried to sleep** |  |  |  |  |  |  |  |  |  |  |  |  |
| Never | 17999 | 15026 | 53.48 | 46.52 | 12109 | 11993 | 48.94 | 51.06 | 5890 | 3033 | 66.39 | 33.61 |
| Rarely | 10767 | 12939 | 47.51 | 52.49 | 5922 | 9376 | 41.55 | 58.45 | 4845 | 3563 | 57.55 | 42.45 |
| Sometimes | 8829 | 13234 | 42.00 | 58.00 | 4291 | 8507 | 35.88 | 64.12 | 4538 | 4727 | 50.97 | 49.03 |
| Most of the time/Always | 2280 | 3809 | 42.45 | 57.55 | 864 | 1949 | 36.74 | 63.26 | 1416 | 1860 | 46.93 | 53.07 |
| **Indicators of aggressive behaviour** |  |  |  |  |  |  |  |  |  |  |  |  |
| **Number of times in a physical fight** |  |  |  |  |  |  |  |  |  |  |  |  |
| 0 times | 25272 | 36148 | 42.13 | 57.87 | 16783 | 27605 | 38.51 | 61.49 | 8489 | 8543 | 51.91 | 48.09 |
| 1 times | 6580 | 4529 | 61.67 | 38.33 | 3299 | 2426 | 62.97 | 37.03 | 3281 | 2103 | 60.43 | 39.57 |
| 2 to 3 times | 4513 | 2644 | 64.02 | 35.98 | 1941 | 1191 | 67.22 | 32.78 | 2572 | 1453 | 62.05 | 37.95 |
| ≥4 times | 3510 | 1687 | 68.40 | 31.60 | 1163 | 603 | 68.75 | 31.25 | 2347 | 1084 | 68.26 | 31.74 |
| **How many times got into fights as result of alcohol** |  |  |  |  |  |  |  |  |  |  |  |  |
| 0 times | 35943 | 41467 | 47.07 | 52.93 | 21564 | 29924 | 42.53 | 57.47 | 14379 | 11543 | 56.03 | 43.97 |
| 1 to 2 times | 2738 | 2553 | 59.39 | 40.61 | 1185 | 1451 | 55.42 | 44.58 | 1553 | 1102 | 62.08 | 37.92 |
| >3 times | 1194 | 988 | 60.97 | 39.03 | 437 | 450 | 60.12 | 39.88 | 757 | 538 | 61.37 | 38.63 |
| **Number of times physically attacked** |  |  |  |  |  |  |  |  |  |  |  |  |
| 0 times | 27400 | 35278 | 43.76 | 56.24 | 17961 | 27068 | 40.04 | 59.96 | 9439 | 8210 | 53.78 | 46.22 |
| 1 times | 5116 | 4240 | 54.95 | 45.05 | 2430 | 2306 | 52.28 | 47.72 | 2686 | 1934 | 57.76 | 42.24 |
| 2 to 3 times | 4053 | 3244 | 56.91 | 43.09 | 1677 | 1587 | 53.33 | 46.67 | 2376 | 1657 | 60.10 | 39.90 |
| ≥4 | 3306 | 2246 | 60.83 | 39.17 | 1118 | 864 | 57.57 | 42.43 | 2188 | 1382 | 62.63 | 37.37 |
