# Supplementary material for: The relationship between psychosocial circumstances and injuries in adolescents: An analysis of 87,269 individuals from 26 countries using the Global School-based Student Health Survey
Source: PLoS Med. 2021 Sep 28;18(9):e1003722. doi: 10.1371/journal.pmed.1003722 (PMC8478259; doi:10.1371/journal.pmed.1003722)
Supplement: Attachment — Submitted filename: Response to reviewers.docx [file pmed.1003722.s004.docx]

Response to comments from the editors and the reviewers

As we found the line numbers are inconsistent in the marked-up version and were changing each time the word document was opened (due to tracked changes), the line numbers are presented in the clean version.

**Requests from the editors:**

1. Abstract: Please structure your abstract using the PLOS Medicine headings (Background, Methods and Findings, Conclusions).

Response: Done as requested. Please see abstract page 2-3, line 31-71.

2. Abstract: Methods and Findings: Please provide summary demographics on the age/ranges of the adolescents. Please quantify the main results presented with both 95% CIs and p values. In the last sentence of the Abstract Methods and Findings section, please describe the main limitation(s) of the study's methodology.

Response: Done as requested. Please see abstract page 2-3, line 36-65.

3. Abstract: Conclusions: Please address the study implications without overreaching what can be concluded from the data; beginning with the phrase "In this study, we observed ..." may be useful. In the second sentence, please temper the wording slightly to avoid causal implications (“...programmes to target...are likely to reduce…”).

Response: We have adjusted the wording slightly to not sound overreaching. Please see abstract page 3 line 67-71.

4. Main text: Please remove the sections “Funding” and “Declaration of interest” from the main text (this information should be included through the manuscript submission system).

Response: The funding and declaration of interest section has been removed as requested.

5. Main text: Please include line numbers with the revised document.

Response: Inserted as requested.

6. Main text: Please ensure all abbreviations are fully defined at first use in the text (for example, DALYs in the Author summary).

Response: We have gone through the text and made sure all abbreviations are fully defined at first use in text. For the quoted example, please see Page 5, Line 123.

7. Author summary: What did the researchers do and find?: Please clarify the third bullet point on protective factors- from the results presentation it seems as if greater numbers of friends, more understanding parents was associated with greater odds of injury, rather than an inverse relationship.

Response: We have adjusted the third bullet point to make it clear that greater number of friends and more understanding parents were not protective factors for having injuries. Please see page 4, line 94-97.

*“We also found that factors which we hypothesised to be protective, such as having close friends or understanding family members or guardians did not have a significant effect on the occurrence of serious injury, apart from in the extreme categories where they were associated with an increase in serious injuries.”*

8. Author summary: What do these findings mean? Please revise the wording of the second bullet point to avoid implying causality (“Targeting...is likely to have the co-benefit of reducing…”).

Response: We have reworded the following sentence to avoid implying causality. Please see page 5, line 109-111.

*“Focusing on psychosocial circumstances of adolescence might have the co-benefit of reducing injury prevalence in adolescence, in multiple country regions and income statuses. “*

9. Throughout: Please place citations within brackets prior to the sentence punctuation. For multiple references within brackets, please do not include spaces.

Response: Noted and changed throughout the text.

10. Methods: Did your study have a prospective protocol or analysis plan? Please state this (either way) early in the Methods section.

Response: We documented our discussion with the co-authors of which variables to include. This is now outlined in the methods; page 7 line 186-189.

*“After discussions between authors, we agreed variables to be included in the analysis, based upon our knowledge of the literature on the relationships between psychosocial variables and violence or other injuries, and the availability of those indicators in the dataset. We documented this prior to starting the analysis.”*

a) If a prospective analysis plan (from your funding proposal, IRB or other ethics committee submission, study protocol, or other planning document written before analyzing the data) was used in designing the study, please include the relevant prospectively written document with your revised manuscript as a Supporting Information file to be published alongside your study, and cite it in the Methods section. A legend for this file should be included at the end of your manuscript.

Response: N/A.

b) If no such document exists, please make sure that the Methods section transparently describes when analyses were planned, and when/why any data-driven changes to analyses took place.

Response: A detailed explanation of the analysis is described in the methods. We have amended this section to include extra detail regarding how independent variables were chosen after receiving the data. Please see page 7-8 lines 183-194.

*“We limited analysis to data collected between 2009-2015 to maximise temporal comparability. Included in the ten core modules, asked by all countries, are questions on social circumstances, alcohol use, drug use, mental health, physical activity, tobacco use, and violence and unintentional injury. After discussions between authors, we agreed variables to be included in the analysis, based upon our knowledge of the literature on the relationships between psychosocial variables and violence or other injuries, and the availability of those indicators in the dataset. We documented this prior to starting the analysis. Questions in the core survey were updated over time, meaning not all countries had data for all variables that the authors considered of potential interest, hence some variables that were listed in the core set, but found to have limited availability, were discarded before agreeing on the final set for inclusion. To be included in this analysis, countries needed to have responses to all questions of relevance.”*

c) In either case, changes in the analysis-- including those made in response to peer review comments-- should be identified as such in the Methods section of the paper, with rationale.

Response: We have adjusted the methods to include a more detailed description of our a priori inclusion of variables and the rationale for discarding some based upon their availability. We did not change any analyses based on the reviewers’ comments. Please see pages 7-8 lines 170-194.

11. Methods: Please ensure that the study is reported according to the STROBE guideline, and include the completed STROBE checklist as Supporting Information. Please add the following statement, or similar, to the Methods: "This study is reported as per the Strengthening the Reporting of Observational Studies in Epidemiology (STROBE) guideline (S1 Checklist)."

The STROBE guideline can be found here: <http://www.equator-network.org/reporting-guidelines/strobe/>

When completing the checklist, please use section and paragraph numbers, rather than page numbers.

Response: Completed checklist attached.

12. Results: Page 11: Please avoid the use of language implying causality. For example, please rephrase sentences such as “Physical activity did not increase the odds of injury.” and “There was no protective effect of…” to avoid the use of causal language.

Response: The language throughout the results section has been changed to try to avoid implying causality. Please see track changes throughout the manuscript.

13. Results: Page 12: In the paragraph describing relationships with aggressive behavior variables, please replace “effect” with “association” in the following sentence (or similar): “...apart from attenuating the relationships between older age and odds of being injured, as well as attenuating the differences between geographical areas.”

Response: The text has been adjusted as requested. Please see page 14, line 322.

14. Discussion: Please present and organize the Discussion as follows: a short, clear summary of the article's findings; what the study adds to existing research and where and why the results may differ from previous research; strengths and limitations of the study; implications and next steps for research, clinical practice, and/or public policy; one-paragraph conclusion.

Response: Adjustments have been done to the discussions. Please see track changes through the discussion. We hope this is sufficient.

15. Discussion: Page 20: Please provide some additional detail for this sentence: “Finally, although several positive associations were seen with the category “other”, lack of detail meant that it was not possible to know what this category contained.”

Response: The category “other” is not an aggregate of variables receiving proportionately lower response rates from participants, rather it represents responses to the questions of “something else” happened to me or similar on the questionnaire. It is not possible to know what it contains therefore. We have added a note to the methods around this as follows (page 8, lines 213-215):

*“The categories of “other” were captured at the time of the survey by respondents documenting that “something else” happened to them. It is not possible to disaggregate this category further.“*

16. Figures and Tables: Please provide titles and legends for all figures (including those in Supporting Information files).

Response: Figures titles and legends have been added to all figures including supporting information files.

Figure 1 – Forest plot showing the multivariable associations (as odds ratio [OR] and 95% confidence intervals [95% CI]) between serious injury occurrence, country level factors, and individual level factors of risky behaviour, contextual factors, protective factors, and indicators of poor mental health.

Figure 2 – Forest plot showing the multivariable association (as odds ratio [OR] and 95% confidence intervals [95% CI]) between serious injury type, country level factors, and individual level factors of risky behaviour, contextual factors, protective factors, and indicators of poor mental health. *Head Injury refers to Concussion or other head or neck injury, was knocked out, or could not breathe

Appendix figure 1: Forest plot showing the multivariable associations (as odds ratio [OR] and 95% confidence intervals [95% CI]) between serious injury occurrence, country level factors, and individual level factors of risky behaviour, contextual factors, protective factors, indicators of poor mental health and aggressive behaviour indicators

17. Table 1 and Table 3: Please include in the legend the explanation for the weighted percentages reported.

Response: Explanation for the weights has been added to the table legends.

The table contains the unweighted number of participants for each category ‘n’ and the weighted percentages. The weighting process accounts for the study design and non-responses. We have added the following to the Table legend:

*“Weights take into account the 2-stage study design and is also adjusted for the non-response rate of participants, see the methods section for a full explanation."*

18. Table 2: Please clarify in the legend that Model 1 is the unadjusted model.

Response: It has been clarified that Model 1 is the unadjusted model in table 2. Please see track changes in row 1 column 2 in table 2.

*“Model 1 (unadjusted model)”*

**Comments from the reviewers:**

As we found the line numbers are inconsistent in the marked-up version and were changing each time the word document was opened (due to tracked changes), the line numbers are presented in the clean version.

Reviewer #1: I confine my remarks to statistical aspects of this paper. The general approach is fine, but I have some issues to resolve before I can recommend publication.

General: Was collinearity investigated? It seems likely with these variables.

Response: Generalised Variance Inflation Factors (GVIF) were used to test for collinearity in this study as the majority of our variables were categorical with >1 degree of freedom. The GVIFs were compared to a threshold of 5^0.5^(≈2.24), which would be comparable to an ordinary VIF threshold of 5. With this threshold none of the variables met the criteria for collinearity.

The following references for this method has been added to the paper:

1. Fox, J. and Monette, G. (1992) Generalized collinearity diagnostics. JASA, 87, 178—183)
2. <https://journals.plos.org/plosone/article?id=10.1371/journal.pone.0093195>

We have now added a statement to this effect to the statistical analysis section and reference 2 and 3, please see page 12, line 242-245 in the clean manuscript:

*“After excluding collinearity between variables using Generalised Variance Inflation Factors, logistic regression was used to estimate associations between outcomes and indicators of psychosocial circumstances, controlling for demographic and country level factors as categorised by the World Bank”*

We have not included the results of these calculations in the paper, but would be happy to add them to the appendices if the reviewer or editor feels that this is necessary.

p. 5  Please also say what % of adolescents are in LMICs. If it is approximately 90%, then the fact that 90% of deaths occur there is not really interesting.

Response: Thanks for this comment. In fact 90% of adolescents live in LMICs and we have corrected our sentence accordingly. Please see page 5, line 116-119 and adjusted text as follows:

*“Moreover nearly 90% of adolescents live in low- and middle-income countries (LMICs) [1]. In 2016, 1.1 million adolescents, globally, died from preventable or treatable causes. Injury is a major cause of death among adolescence [2], with most of these deaths occurring in LMICs [3].”*

        Give numbers of deaths for the various causes.  To say something is a "leading cause" isn't very meaningful; even if deaths are very rare, something has to be the leading cause.

Response: We have adjusted the sentence. Please see page 5, line 118-119 and adjusted text as follows:

*“Injury is a major cause of death among adolescents [2], with most of these deaths occurring in LMICs [3].”*

p. 10 How did you ``adjust for study design"?  That you used certain variables is not sufficient information. Did you use robust standard errors? A multilevel model? GEE? Something else?

Response: Study design was adjusted for by the method indicated in the GSHS user guide (<https://www.cdc.gov/gshs/pdf/gshs-data-users-guide.pdf>) to take into account the 2-stage sample design and the weighting process. Information on this has been added to the method section of the paper.

Please see page 12 line 246-260 and adjusted text as follows:

*“We adjusted for study design by using the survey’s stratum, weights and primary sampling unit variables (PSU). These latter variables were used to account for selection of schools and classrooms, non-responding schools and students, and population distribution by grade and sex, as detailed in the GSHS Questionnaire users guide [46]. The study weights (W) were calculated using the following equation; W=W1xW2xf1xf2xf3. W1, the inverse probability of selecting each school; W2, the inverse probability of selecting each classroom; f1 and f2, the non-response adjustment factors at school-level and classroom-level, respectively; and f3, a post-stratification adjustment factor calculated by sex within grade. The stratum was assigned sequentially starting at schools with the largest enrolment through to schools with the smallest. The PSU was assigned sequentially starting with the classes within the schools with the largest enrolment of students and continuing through those with the smallest enrolment. The weights, stratum and PSU were provided with the GSHS data. However, to ensure that school assignments were unique between countries, the given stratum assignment was adjusted by adding a 3-digit prefix that specified which country the schools were in.“*

          It is good that you used forced entry.

Response: Thank you. We are happy to hear you agree with this approach.

         Rather than list a version of RStudio, it would be good to list a version of R.  RStudio is just an editor.  (After all, you don't list which version of Word you used).

Response: Thanks for this. R version 3.6.1 has been added to statistical analysis section. Please see page 13, line 274-275 and adjusted text as follows:

*“We used R version 3.6.1 (www.rstudio.org) to analyse the data, and two-tailed p-values <0.05 were considered statistically significant. Complete case analyses were used.”*

Figures:  The font is too small to be readable on a printout. One way to help fix this is to merge the two left-most columns and simply indent the variable names by a few spaces.  I'm not sure if that will be enough, but it's a start.

Response: The figures have been adjusted as suggested. Please see updated figures attached.

Peter Flom

Reviewer #2:

As we found the line numbers are inconsistent in the marked-up version and were changing each time the word document was opened (due to tracked changes), the line numbers are presented in the clean version.

This study examined psychosocial factors associated with serious injury occurrence, mechanism, and type in adolescents. Its sampling methodology, measurement of the variables, and writing are appropriate.

My concerns about the article mostly are:

1. Although there are many correlations in this study the theoretical support of the manuscript is lacking, what are the motivations of the study based in the behavioral and psychology literature? Lacking theoretical perspectives, the model itself cannot answer the why manifests these associations.

Response: We developed the concept for the study based on our knowledge of the literature. In particular using the definition of psychosocial as “pertaining to the influence of social factors on an individual's mind or behavior, and to the interrelation of behavioral and social factors” (OED, 2012), and previously shown associations (in small, usually individual setting studies) between dependent (injury) and independent (psychosocial) variables.

We grouped the variables into categories in order to aid interpretation of the results. This was also based on discussions amongst the authors and informed by knowledge of the scientific literature about causes of injuries in LMICs, please see references 3, and 12-35. However, our data are cross-sectional hence we can only present associations and cannot prove causation or indeed the reasons for these associations. We considered using techniques such structural equation modelling, causal influence models or mediation analysis, however we do not consider these models are reliable enough to prove causation with cross sectional data where reverse causality cannot be ruled out. To report results from them might introduce spurious assertions of causality. Unfortunately, there are no longitudinal data available at the moment to try to understand causation using quantitative means. That said, this is the largest multi-country study to date showing strong associations between psychosocial variables and injuries, their type, and mechanisms, and we have discussed the potential directionality of the responses in the discussion, based on our knowledge of the literature in this area. Our manuscript highlights these variables as an areas for concern in LMICs, where injuries are highly prevalent amongst adolescent populations.

2. Describe about psychosocial circumstances is not clear, please clarify.

Response: We have elaborated on psychosocial circumstances in the methods of the abstract. Please see page 2, line 37-45 in the clean manuscript:

*“We used logistic regressions to estimate associations between prevalence of serious injuries, injury type (body system affected), or injury mechanism (cause of injury) and psychosocial factors (factors that relate to a person socially, or their thoughts or behaviour, or the interrelation between these variables). Psychosocial factors were categorised, based on review of the literature, author knowledge and discussion amongst authors. The categories were; markers of risky behaviour (smoking, alcohol, drugs or psychical activity), contextual factors (hunger, bullying or loneliness), protective factors (number of friends or supportive family), and markers of poor mental health (planned or attempting suicide or being too worried to sleep).”*

We also recognise that there were some other variables which could have been included in the model, but we limited the numbers of variables included by the above process to prevent overfitting of the model and dependence between explanatory variables.

We have now better-defined psychosocial circumstances in the methods. Please see page 9, line 217-231 and text as follows:

*“Variables were selected for inclusion based on their availability in the surveys, and hypothesised relationship with the outcome variables. Their inclusion was agreed on discussion amongst authors. For clarity of interpretation of the results, we divided the variables into overarching categories (see panel 1) with all conceptualised as psychosocial factors – defined as factors that relate to a person socially, or their thoughts or behaviour. Indicators of psychosocial circumstances contains markers of risky behaviour, with variables hypothesised to be associated with risk taking or an increased risk of injury (e.g. smoking, alcohol, drug use, or physical activity); contextual factors, with variables which could suggest the respondent lives in an environment which makes them vulnerable, hence more susceptible to injury (e.g. going hungry, bullying, or feeling lonely); [41]: protective factors, with variables which may suggest a nurturing environment with social support which might lower risk of injury occurrence (e.g. number of friends or supportive family); and indicators of poor mental health with variables previously identified in the General Health Questionnaire for detecting minor psychiatric morbidity (e.g. planned or attempting suicide, or being too worried to sleep) [41, 42].*

We defined feeling lonely as a contextual factor as we thought it best reflected the actual social state of individuals. Although, we do recognise that feeling lonely may also indicate a state of mind – and may have been alternatively placed in markers of poor mental health - rather than an actuality. We have added a section about this to the limitations. Please see page 22, line 553-559 and adjusted text as follows:

*“Also, we recognise that our definition of psychosocial factors is not necessarily the only way of categorising these variables. For example, we categorised feeling lonely along with other contextual variables, considering it as being indicative of the individual’s social status. However we do acknowledge that feeling lonely may indicate a state of mind rather than an actuality, and hence could be considered a marker of poor mental health. Nevertheless, the categorisation of variables does not influence their relationship with the outcome variables”*

We hope this is clearer now.

3. Family and school factors are important contextual variables, please answer why did not included them in analysis.

Response: The major factor that we felt would have associate with injury was whether or not the respondent attended school. However, all the participants were in school, hence we were not able to compare injuries who did compared with who didn’t attend school. We have already added this to the limitations. Please see page 23, line 560-563:

*“Additionally, the nature of the survey methodology meant that respondents were all in school, which itself may be protective against injuries and poor mental health [35]. A broader household survey which captures responses from adolescents who are not in school may have found stronger associations between psychosocial factors and injuries.”*

Additionally, we could only include selected variables which were present in the original questionnaire and asked in the multiple countries included in this study. We also aimed to avoid overfitting of the model by limiting the number of variables included in our analyses – with methods to do this as described above - There were three potential family-related questions in addition to the one selected for use: “During the past 30 days, how often did your parents or guardians understand your problems and worries?” These were as follows. 1. During the past 30 days, how often did your parents or guardians really know what you were doing with your free time? 2. During the past 30 days, how often did your parents or guardians go through your things without your approval? 3. During the past 30 days, how often did your parents or guardians check to see if your homework was done? However, on discussion, we felt that the variable included was the best at representing a supportive home environment. Please also refer to the above response on overfitting the model and text as follows (page 7-8, line 183-194):

*“We limited analysis to data collected between 2009-2015 to maximise temporal comparability. Included in the ten core modules, asked by all countries, are questions including on social circumstances, alcohol use, drug use, mental health, physical activity, tobacco use, and violence and unintentional injury. After discussions between authors, we agreed variables to be included in the analysis, based upon our knowledge of the literature on the relationships between psychosocial variables and violence or other injuries, and the availability of those indicators in the dataset. We documented this prior to starting the analysis. Questions in the core survey were updated over time, meaning not all countries had data for all variables that the authors considered of potential interest, hence some variables that were listed in the core set, but found to have limited availability, were discarded before agreeing on the final set for inclusion. To be included in this analysis, countries needed to have responses to all questions of relevance. “*

4. This multi-stage random sampling design, but statistic analysis did not account for multiple stages of sampling stratification and clustering, which may lead to some errors. I suggest conduct re-analyze using complex sample analysis methods.

Response: Our method did take into account multistage random sampling stratification. The sampling process was incorporated in our svydesign object in R, and took into account the probability of picking a school, classroom and also adjusted for non-responses and other factors that were incorporated in the survey design. We designed this method from using the Survey’s analysis user guide, and this approach has been used in other publications*. See a more detailed explanation in the statistical part of the methods section line 253-263 as per response to reviewer 1’s comment on this issue.

* Han, L., You, D., Gao, X., Duan, S., Hu, G., Wang, H., Liu, S. and Zeng, F., 2019. Unintentional injuries and violence among adolescents aged 12–15 years in 68 low-income and middle-income countries: a secondary analysis of data from the global school-based student health survey. The Lancet Child & Adolescent Health, 3(9), pp.616-626. DOI:https://doi.org/10.1016/S2352-4642(19)30195-6

5. The discussion is merely a reiteration of results and comparison with some published studies. In-depth explanation of the findings is necessary.

Response: We have tried to elaborate more on our findings in the discussion, however, to report an in-depth explanation of the findings which goes beyond what is stated in the literature would be to introduce spurious causality, and we are keen to avoid over interpretation of our results. Please see track-changes throughout the discussion. We have outlined some examples below.

Page 18, Line 415-419:

*“Interestingly, factors which we initially hypothesised would be associated with a more supportive psychosocial environment for example, having a greater number of friends, or more understanding parents or guardians - were not protective of injury risk. Although this was initially surprising and went against our hypothesis, in some settings friends or family could be supportive of criminogenic behaviour leading to increased exposure to injuries [47].”*

Page 18, Line 436-441:

*“However, in our model, bullying remained a strong independent predictive factor of odds of injury even when controlling for variables indicating poor mental health, suggesting that it is not solely acting through these variables. Moreover, bullying could lead to social exclusion and drive risk taking behaviour – and thence injuries - as a compensation mechanism to overcome the stress of bullying and social exclusion [48-50].”*

6. The manuscript appeared messy in many associations describes, proper organization and concentration are necessary.

Response: As a preference, we presented the findings in both the text and in tables as some readers prefer this, however if the editor would like us to reduce the text and refer more to the tables and figures we are happy to. We have additionally read through and edited the results to ensure that they are presented consistently in the same order in the text and the tables, to aid with clarity.

Reviewer #3:

As we found the line numbers are inconsistent in the marked-up version and were changing each time the word document was opened (due to tracked changes), the line numbers are presented in the clean version.

This is a well written paper on important, if not complex, analyses to further understanding on how best to prevent injuries among youth. It is strongly presented as is. Just some thoughts are offered and only minor recommended revisions.

Thank-you for these positive comments

While appreciating the numerous variables to explain and the need for appendices and referral to other publications, one aspect important to explain within this manuscript is the age groupings. This does not appear to be addressed (besides the need for upper and lower cut-offs and lack of age range). Was this a grouping agreed by authors and/or how was this determined?

Response: This was a limitation set by the survey questions in the GSHS, where groupings varied between countries and did not have specific cut offs. We combined age ranges to ensure harmonisation across the surveys, but we were unable to present these in a more granular way than we have done. For example, in the Indonesia survey the upper and lower cut offs were ’16 years and older’ and ‘11 years old or younger’. That said the surveys were done in secondary school age children hence the majority were between the ages of 13 and 17.

We have added this as a limitation Page 23, line 574-579 in clean version of the manuscript:

*“Descriptions of age groupings varied between countries and did not have specific cut offs. We combined age ranges to ensure harmonisation across the surveys, but we were unable to present these more granularly. For example, in the Indonesia survey the upper and lower cut offs were ’16 years and older’ and ‘11 years old or younger’. Nevertheless, the surveys were done in secondary school age children hence the majority were between the ages of 13 and 17.”*

In different country socio-cultural contexts, there could perceivably be large differences between 14 years and 15 years for example in terms of exposures, particularly by gender; for example, acceptability of young males versus females being out unchaperoned at night, taking part in contact sports, official working age or legal age for riding motorised vehicles (such as mopeds); appreciating there are controls by country. While the limitations section was particularly strong and appreciated, the lack of ability to measure or control for such other types of 'exposures' to injury risk might also be acknowledged.

Response: Unfortunately we did not have age as a continuous variable and we could therefore not tease out the more subtle age differences in risk. We have added a few sentences on this to the limitation section. Please see page 23, line 580-584 and adjusted text as follows:

*“That the dataset did not have age as a continuous variable meant that it was not possible to tease out more subtle age differences in exposure that may occur on crossing an age milestone. For example driving motorised vehicles or taking part in contact sports may increase on passing a country-determined age threshold. However, for females, going out unescorted may decrease with certain coming of age thresholds.”*

The addition of the aggressive behaviour indicator variables did not (essentially) change the main model and all held significant associations. Did you explore the proportions of variance explained by the models and might these additions have increased the explained variance?

Response: We did not originally explore this, but have done so below:

|  | Sum of studentised residuals squared | AIC |
| --- | --- | --- |
| Model 1 | 101354.10 | -202500 |
| Model 2 | 96413.88 | -192600 |

Although the addition of aggressive behaviour did reduce the studentised residuals (a marker of deviance), it also increased the AIC. So although the added variables explained some of the variance in the model, this was not enough to counteract the added complexity to suggest it was a better model.

We have not added this to the manuscript, as we feel it may over complicate the already complex results. But if the reviewer or editor feels that it would strengthen it, we would be happy to add it to the results section.

Thinking through the curiosity of lack of anticipated protective factor associations, I wonder if this might relate to the dominant low-to-middle income country participation, with most of the associated research from high-income countries, as acknowledged by the authors. It is possible there is more to understand about the full wording of the questions and language translation (including whether translations were both from English and back to English?). Just "having more" friends and "supportive parents" out of context may be blurred as protective within (sub)cultures with strong criminogenic tendencies. That is, more friends and parents supportive of criminogenic behaviours would then be another form of increased exposure to injury and would cancel out expected protective associations. Within offender management literature, reducing contact with criminogenic family and friends is one of the proven success factors to reduce reoffending (see for example, evidence-based work led by James Bonta and Donald Andrews on the Risk-Need-Responsivity model).

Response: Thanks for pointing out this interesting point. We have already touched upon that friends may be of the wrong sort, but we have added more depth to this point in the discussion and cited the reference given. Please see page 18, line 415-419 and adjusted text as follows:

*“Interestingly, factors which we initially hypothesised would be associated with a more supportive psychosocial environment for example, having a greater number of friends, or more understanding parents or guardians - were not protective of injury risk. Although this was initially surprising and went against our hypothesis, in some settings friends and family could be supportive of criminogenic behaviour leading to increased exposure to injuries [47].”*

Minor

Abstract: Citing unintentional injury statistics here, and then again in the Introduction, made it not entirely certain whether these were the main focus rather than both intentional and unintentional until reading the Methods. This could be more explicit.

Response: We are focusing on intentional and unintentional injuries. We have changes this wording throughout the text. Please see page 2, line 31-33 in the abstract:

*“Large numbers of adolescents die globally each year from preventable or treatable causes, with injuries (intentional and unintentional) being the leading cause of these deaths.”*

And page 7, line 162-167 in the introduction:

*“We aimed to ascertain associations between psychosocial factors potentially associated with the occurrence of any serious injury (intentional and unintentional), the most serious injury type, and the most serious injury mechanism using data from the Global Schools-Based Student Health Survey, and to compare associations across geographical regions and by level of country development.”*

Abstract: why is "<37%" included rather than the actual rounded %?

Response: This has been corrected to the correct number with decimals. Please see page 2 line 50-52 and adjusted text as follows:

*“The weighted prevalence of a serious injury in the last 12 months was 36.33%, with the rate being higher in low-income countries compared to other income countries (48.74% vs 36.14%) and amongst males compared to females (42.62% vs 29.87%).”*

Last point on p4, "All available evidence points to psychosocial factors such as bullying, drug taking, or poor mental health are strongly associated…",  should be "as strong associated"?

Response: The sentence has been adjusted accordingly. Please see adjusted text page 5, line 106-108:

*“All available evidence points to psychosocial factors such as bullying, drug taking, or poor mental health are as strongly associated with occurrence of injury and consistent across income status and geographical regions. “*

Introduction first sentence is missing a reference.

Response: Thanks for noticing this. There correct reference has been added.

Methods: Noting whether self-completion of the survey was at school in all contexts or perhaps independently on-line for example would add further contextual understanding.

Response: The survey was completed at schools. We have added this to the methods. Please see page 7 line 175-176:

*“Surveys are self-completed by students at their schools.”*

Results: There was inconsistent reporting of the p value findings in terms of conventions of <0.05, <0.01, <0.001, to reporting "=" to rounded values. I noted only one case where p=0.05 was appropriate (last line page 12), otherwise it would be less distracting to consistently apply the 'less than' convention.

Response: We have now reported the p-values consistently in terms of conventions in the tables and throughout the text.

Page 11 paragraph 4, "Considering markers of risky behaviour, ever having used drugs was significantly associated with injury (OR 2.08, 95% CI 1.73 - 2.49, p< 0.001), but the relationships between smoking or alcohol and injury were not consistent": use of "were not consistent" is ambiguous as to whether this refers to the same as 'ever used drugs' or consistent between alcohol and smoking, rather than a lack of dose-response type associations for these variables - which I assume this was meant to convey. Perhaps just "were inconsistent" would be better here.

Response: Sorry for the confusion, we meant there wasn’t a consistent dose response. Please see adjusted text page 13 line 293-298.

*“Considering markers of risky behaviour, the relationships between smoking or alcohol and injury were inconsistent, with some exposures being associated with increased odds of injury, whilst most were not. However, ever having used drugs was significantly associated with injury (OR 2.08, 95% CI 1.73 - 2.49, p< 0.001).”*

There is also some inconsistency in explaining that the cited odds examples in brackets are for the highest dose/level only for some of the variables with multiple levels for which all levels were significant (compared to the reference value). For example, this is stated as an example "for most of the time or always" on last line page 11, but not elsewhere. While it might be somewhat repetitive to state this on every occasion, an overarching statement could be made that the highest dose/level statistics are included as examples in brackets, when applicable (adding "e.g.," to the bracketed examples).

Response: A statement has already been added to text. Please see page 13 line 272-273.

*“Results are presented in the text for the odds of the highest compared to lower exposure level of each variable, unless otherwise stated.”*

Discussion: there were a few typos to spell check in this section (e.g., top page 16 "bulling").

Response: Thank you for noticing these typos. They have been adjusted accordingly. Please see track changes throughout the discussion.

Top of page 18: I was anticipating a comment about 'but not for considering suicide only'?

Response: We were puzzled by the lack of relationship seen and have now added a sentence about this to the discussion as follows page 21 line 525-529:

*“That we found that attempting suicide but not considering or planning suicide was associated with increased odds of injury was interesting and not due to there being too few people who had considered suicide to detect an effect. This deserves further exploration in future studies.”*

Then to end the paper, this somehow seemed to lack a strong conclusion section, despite the many strength and insights on offer. The end paragraph was more focused on implications but could have offered some more summary statements also. Note that 'SDGs' is not first explained or referenced before use here.

Response: The sustainable development goals (SDGs) have now also been mentioned in the introduction. We have also tightened up the conclusion as follows please see page 24 line 609-615.

*“In this large multi-country study we have shown that the association between injury and indications of psychosocial circumstances is complex. Serious injuries were strongly associated with multiple factors across categories of markers of risky behaviour, contextual factors, protective factors, indicators of poor mental health, and aggressive behaviour. These findings enforce the view that addressing injuries will require considering multiple of the SDGs, not limited to the specific injury SDG, 3.6. Multicomponent interventions to improve psychosocial circumstances will likely be needed to substantially reduce injury prevalence. ”*

Note also that the copies of the figures within the PDF were almost illegible (very blurry). In reviewing, rather than persevere to try to check all of these, the main focus was on the tables (besides noting the patterns that are helpful to have visually given the many numbers in lengthy tables).

Response: We have improved the resolution of our figures. Please see attached.
