# Supplementary material for: The relationship between psychosocial circumstances and injuries in adolescents: An analysis of 87,269 individuals from 26 countries using the Global School-based Student Health Survey
Source: PLoS Med. 2021 Sep 28;18(9):e1003722. doi: 10.1371/journal.pmed.1003722 (PMC8478259; doi:10.1371/journal.pmed.1003722)
Supplement: Attachment — Submitted filename: Response to editors 040721.docx [file pmed.1003722.s005.docx]

**Requests from Editors:**
All line numbers indicating where revisions are made are according to the clean version of the manuscript.

1. Abstract: Background: If possible, please be more specific when describing “Large numbers of adolescents…”

Response: We have adjusted the text as follows, please see page 2 line 31-32;

“Over a million adolescents die globally each year from preventable or treatable causes, with injuries (intentional and unintentional) being the leading cause of these deaths”

2. Abstract: Line 46: Please clarify what is meant by “geography” in terms of the country-level factors for which you adjusted.

Response: We have clarified what we mean by geography in the abstract. Please see page 2 line 46-48.

“Models were adjusted for country factors (geographical area and income status both using World Bank Classification), demographic factors (age and sex) and factors to explain the survey design.”

3. Abstract: Conclusions: Line 68: We would suggest “...but we note the relationships are likely to be complex and our findings do not inform causality.” or similar, to clarify.

Response: Thank you for your suggestion. We have adjusted the text accordingly. Please see Page 3, Line 68-70.

“We observed strong associations between serious injury and psychosocial factors, but we note the relationships are likely to be complex and our findings do not inform causality.”

4. Abstract: Line 69: We suggest changing “indicate” to “suggest” and at line 70-71, we suggest changing to read “...in particular those concentrating on reducing bullying and drug use and improving mental health.”

Response: We have adjusted the text as suggested. Please see Page 3, Line 70-72.

“Nevertheless, our findings suggest that multifactorial programmes to target psychosocial factors might reduce the number of serious injuries in adolescents, in particular those concentrating on reducing bullying and drug use and improving mental health.”

Additionally, to make the findings and recommendations of this study clearer, we have also adjusted the Author’s summary. Please see Page 5, Lines 110-114.

- “Our findings suggest that multifactorial programmes to target psychosocial factors might reduce the number of serious injuries in adolescents across different countries and contexts.
- In particular, concentrating on reducing bullying and drug use and improving mental health could reduce number of serious injuries among adolescents in the future.“

5. Introduction: Line 155: We suggest “..about each adolescent’s health…” or similar.

Response: The text has been adjusted to reflect the editor’s suggestion please see Page 6, Line 156-159.

“The Global School-based Student Health Survey (GSHS) is an ongoing collaborative surveillance project between WHO and the Centers for Disease Control (CDC) [39, 40] involving 101 countries and consisting of a series of questions about each adolescent’s health and living circumstances.

6. Methods: Line 186: Please change to “...we agreed on variables…” or similar.

Response: The text has been adjusted please see Page 7, Line 189-192.

“After discussions between authors, we agreed on variables to be included in the analysis, based upon our knowledge of the literature on the relationships between psychosocial variables and violence or other injuries, and the availability of those indicators in the dataset.”

7. Methods: Line 191-193: Thank you for the clarification regarding the planning of your analyses. Please mention in the text the variables that were intended to be included, but could not be included due to limited availability: “...hence some variables that were listed in the core set, but found to have limited availability, were discarded before agreeing on the final set for inclusion.”

Response: The variable which was excluded due to limited availability is now mentioned on Page 8 Line 193-198.

“Questions in the core survey were updated over time, meaning not all countries had data for all variables that the authors considered of potential interest, hence some variables that were listed in the core set, but found to have limited availability, were discarded before agreeing on the final set for inclusion – therefore “Have you ever had sexual intercourse?” was excluded from the analysis due to limited availability.”

8. Methods: Line 218-219: Please change to “Their inclusion was agreed on through discussion amongst authors.” or similar.

Response: We have adjusted the text, please see Page 9, Line 222-223.

“Their inclusion was agreed upon through discussion amongst authors.”

9. Methods: Early in the methods, please add the following statement, or similar, to the Methods: "This study is reported as per the Strengthening the Reporting of Observational Studies in Epidemiology (STROBE) guideline (S1 Checklist)."

Response: We have added the suggested text please see Page 7, Line 185-186.

“This study is reported as per the Strengthening the Reporting of Observational Studies in Epidemiology (STROBE) guideline (S1 Checklist).”

10. Methods: Please provide the name(s) of the institutional review board(s) that provided ethical approval for the study.

Response: An ethical statement has been added to the methods. Ethical approval was given from each country’s local ethics government committee, as stated in the survey’s website. Please see ethical statement page 13 line 282-288.

“Ethical statement

Data collection was approved by each country’s local ethics governance committee. Permissions to conduct the survey in each school were obtained from heads of the selected schools and classroom teachers, written or verbal consent to take part in the survey was given by students or their parents, and surveys were completed anonymously ([www.cdc.gov/GSHS/](http://www.cdc.gov/GSHS/) and www.who.int/ncds/surveillance/gshs/). This study only used pre-existing anonymous survey data, and no further ethical approval deemed necessary.”

11. Results: Line 297-298: Please consistently provide the results, with 95% CIs and p values for all major results described in the text, even if not statistically significant (“Physical activity was not associated with an increased odds of injury.”

Response: This has been corrected. Please see changes throughout the results section.

12. Results: Throughout this section (as well as any tables/figures) please report exact p values, unless p<0.001.

Response: The exact p-values are now given in all the tables (see highlighted numbers in the tables) and track changes throughout the results section unless they are p<0.001.

13. Results: Line 334: Please change this to “...ever having used drugs…” and please also make this change at line 365.

Response: The text has been adjusted as suggested, please see Page 15, Line 350-353 and Page 16 line 380-385.

“Considering markers of risky behaviour, ever having used drugs was significantly associated with increased odds of broken bone/dislocated joint (OR 1.73, 95% CI 1.37 - 2.18, p<0.001), cut or stab (OR 1.73, 95% CI 1.20 - 2.49, p<0.01), or gunshot (OR 3.43, 95% CI 1.63 - 7.20, p<0.001).”

“Ever having used drugs was associated with greater odds of MVA (OR 1.52, 95% CI 1.11 - 2.08, p<0.05); being hit or injured by a falling object (OR 1.88, 95% CI 1.18 - 3.00, p<0.01); or attacked, abused or fighting (OR 2.11, 95% CI 1.31 - 3.41, p<0.01). Higher levels of physical activity were associated with falls (OR 1.47, 95% CI 1.18 - 1.83, p<0.001 for ≥3 days per week compared with none).”

14. Results: Line 366: Please clarify if this is meant to indicate “...being injured by something falling…” or similar.

Response: We have clarified in the text that it is meant to indicate being hit or injured by a falling object. Please see Page 16, Line 380-385. We hope this is cleared now. Please see adjusted text in previous comment.

15. Discussion: Line 402: We suggest changing to “...these relationships may be consistent across world regions.” or similar.

Response: We have changed the text as suggested. Please see Page 17-18, Line 417-419.

“Controlling for geographic region and income status of countries did not change the results, suggesting that these relationships may be consistent across world regions.”

16. Discussion: Line 413: We suggest changing to “... suggests that improving the psychosocial environment for adolescents across multiple country settings could have benefits for prevention of injury.”

Response: The text has been adjusted as suggested. Please see Page 18, Line 428-431.

“That we have found the relationship between these variables is not substantially nor significantly adjusted by adding country income status or geographical area to the model, suggests that improving the psychosocial environment for adolescents across multiple country settings could have benefits for prevention of injury.”

17. Discussion: Line 417: Please change “...were not protective of…” to “...were not associated with…”

Response: We have adjusted the text as suggested. Please see Page 18, Line 432-434.

“Interestingly, factors which we initially hypothesised would be associated with a more supportive psychosocial environment for example, having a greater number of friends, or more understanding parents or guardians - were not associated with injury risk.”

18. Discussion: Line 449-451: Please revise to: “... being struck by an object…”

Response: We have revised the text to reflect the variable more accurately. Please see Page 19, Line 466-468.

“Moreover, drug use was significantly associated with injury types of broken bone or dislocated joint, being cut or stabbed, or gunshot wound and mechanisms of being hit or struck by a falling object or being attacked, abused or fighting.”

19. Figure 1 and Figure 2: In the left column, some of the category names are cut off (e.g. Number of days smoked in...); Number of days of alcohol in…). Please add p values to the figure, in addition to the 95% CIs or please indicate these are presented in the tables. Please note in the legend the variables adjusted for in the analysis, or if these are the results of the unadjusted analysis. If adjusted, please also ensure that the results of the unadjusted analysis are presented in the manuscript (at least in a table).

Response: Figure 1 and Figure 2 have been edited so all the names are now showing in full. Also we have added to the legend what this analysis was adjusted for, and that the p-values can be found in table 2 and 4. Please see track changes Page 25-26 Line 635-661. As regards to the unadjusted analysis please see response to comment 21.

“Figure legends

Figure 1 – Forest plot showing the multivariable associations (as odds ratio [OR] and 95% confidence intervals [95% CI]) between serious injury occurrence and psychosocial circumstances, adjusting for country level factors and demographic characteristics, but not for aggressive behaviour indicators. Adjustment for non-response rate of participants was done as described in the methods. P-values can be found in Table 2, Model 1.”

“Figure 2a – Forest plot showing the multivariable association (as odds ratio [OR] and 95% confidence intervals [95% CI]) between serious injury type (broken bone or dislocated joint, cut or stab wound, head injury* and gunshot wound) and markers of psychosocial circumstances, adjusting for country level factors and demographic characteristics, but not for aggressive behaviour indicators. Adjustment for non-response rate of participants was done as described in the methods. P-values can be found in Table 4.

*Head Injury refers to the variable “Concussion or other head or neck injury, was knocked out, or could not breathe”

“Figure 2b - Forest plot showing the multivariable association (as odds ratio [OR] and 95% confidence intervals [95% CI]) between serious injury type (bad burn, poisoned or took too much of a drug and something else happened to me) and markers of psychosocial circumstances, adjusting for country level factors and demographic characteristics, but not for aggressive behaviour indicators. Adjustment for non-response rate of participants was done as described in the methods. P-values can be found in Table 4.”

“Appendix figure 1: Forest plot showing the multivariable associations (as odds ratio [OR] and 95% confidence intervals [95% CI]) between serious injury occurrence and markers on psychosocial circumstances, adjusting for country level factors, demographic characteristics, and aggressive behaviour indicators. Adjustment for non-response rate of participants was done as described in the methods. P-values can be found in Table 2, Model 2.”

20. Figure 2: Please consider splitting the table up, or reformatting, as the font size seems difficult to read when sized to fit on a single page.

Response: Figure 2 has been split into two figures Figure 2a and Figure 2b to make it easier for the reader.

21. Table 2, Table 4, Table 5: In the legends, please note the variables adjusted for in each model, and for the analyses in Tables 4 and 5, please also include the unadjusted results. Please present the actual p value, except where p<0.001. Instead of NS, please indicate the p values.

Response: We have made the variables which are included in the model clearer in the table headings. Additionally all the actual p-values have been reinserted in the tables. Please see highlighted text in the tables. We have also updated the legends to note the variables adjusted for in the model.

22. References: Please check each reference for “Vancouver” style (see our guidelines at: <https://journals.plos.org/plosmedicine/s/submission-guidelines#loc-references>), and please check all journal title abbreviations. For example, in reference 48, The British Journal of Educational Psychology should be Br J Educ Psychol.

Response: We have gone through the references and checked the abbreviations. Please see track changes throughout the reference section.


Comments from Reviewers:

Reviewer #1: The authors have addressed my concerns and I now recommend publication.


Reviewer #2: The authors adequately addressed my concerns. I think this manuscript may be accepted.


Reviewer #3: The authors have thoroughly addressed the reviewer comments.

I suggest the statement in their first reply to Reviewer #1, that "none of the variables met the criteria for collinearity" is added to the manuscript. This in part is important to the findings regarding explained variance. I personally think the statistics and comments made in this regard are useful to the reader, and could be stated within the text (rather than requiring an additional table), but am happy to defer to the Editor.

Response: None of the variables met the criteria for collinearity has been added to the manuscript as requested. Please see page 12 line 246.

“None of the variables met the criteria for collinearity.”

Some minor grammatical revisions might also be addressed in copyediting
48-50 "from lower-middle income country (81.93%), or from East Asia and the Pacific (66.83%)" - use of "or" is incorrect as the percentages total more than 100.

Response: We have changed the wording, please see page 2 line 48-50.

*“*The weighted majority were 14-15 years old (45.88%), male (50.70%), from a lower-middle income country (81.93%), and from East Asia and the Pacific (66.83%).”

88 "We have quantified..." - delete "have" for consistent grammatical tense with the other points.

Response: We have deleted ‘have’. Please see page 4 line 89-91.

“We quantified the association between psychosocial circumstances and serious injury occurrence, mechanism and type in adolescents, taking into account country as well as individual factors that could affect the relationship.”

109 "Focusing on psychosocial circumstances of adolescence might have the co-benefit of reducing injury prevalence in adolescence,..." is non-specific, rather suggest adding context, as referred to elsewhere of "multifactorial programmes".

Response: We have adjusted this text as follow and added another bullet point. Please page 5 line 110-114

“Our findings suggest that multifactorial programmes to target psychosocial factors might reduce the number of serious injuries in adolescents across different countries and contexts.”

“In particular concentrating on reducing bullying and drug use and improving mental health could reduce number of serious injuries among adolescents in the future.”

Table legend "Weights take into account the 2-stage study design and is also adjusted for the non-response rate of participants, see the methods section for a full explanation." Change "is also adjusted" to "also adjust"?

Response: This has been edited. Please see all the table legends.


Any attachments provided with reviews can be seen via the following link:
